# A Novel 1,3,4-Oxadiazole-Based Fast Dual-Mode Sensor for the Detection of Iron and Lead Ions: Synthesis and Photophysical Properties

**DOI:** 10.1021/acsomega.6c03258

**Published:** 2026-06-10

**Authors:** Abdulrahman Altay, Hamza Karakuş, Ebru Bozkurt, Muhammet Yildirim, Murat Olutas

**Affiliations:** † The Hazardous Chemical & Waste Department, Ministry of Environment and Climate Change, Doha 22445, Qatar; ‡ Technology Transfer Application and Research Center, 52942 Bolu Abant İzzet Baysal University, Bolu 14030, Turkey; § Program of Occupational Health and Safety, Vocational College of Technical Sciences, 37503 Atatürk University, Erzurum 25240, Turkey; ∥ Department of Nanoscience and Nanoengineering, Graduate School of Natural and Applied Sciences, Atatürk University, Erzurum 25240, Turkey; ⊥ Department of Chemistry, Faculty of Arts and Sciences, Bolu Abant İzzet Baysal University, Bolu 14280, Turkey; # Department of Physics, Faculty of Arts and Sciences, Bolu Abant İzzet Baysal University, Bolu 14030, Turkey

## Abstract

In this study, a novel 1,3,4-oxadiazole aniline derivative (**ODADA**) was synthesized via a multistep synthetic method, and its structure was confirmed by FT-IR, ^1^H NMR, ^13^C NMR, and ESI-MS results. The quantum chemical calculation was also conducted in detail, including geometry, electronic structure, charge distribution, and potential intramolecular charge transfer (ICT) behavior of **ODADA**. In addition, photoluminescence (PL) studies on solid-state samples were carried out, and PL emission maxima were observed between 511 and 543 nm. The photophysical properties of **ODADA** in the solution state have also been investigated by UV–vis absorption and fluorescence spectroscopy, and it emits deep blue fluorescence with the obtained absorption and emission maxima at 321–339 nm and 383–468 nm, respectively, and the fluorescence quantum yields (Φ) were found to vary between 0.01 and 0.42 in different solvents. Finally, sensor properties of **ODADA** were investigated for the selective determination of heavy metal ions. The molecule **ODADA** was observed to exhibit a significant colorimetric response to Pb^2+^ ions and a strong fluorometric response to Fe^3+^ ions. Spectroscopic analyses revealed a limit of detection (LOD) of 0.06 μM and a linear range of 0–150 μM for Pb^2+^ and an LOD of 0.10 μM and a linear range of 0–120 μM for Fe^3+^. Selectivity tests showed that the molecule exhibited superior specificity toward other metal ions. However, real sample tests using commercial hair dye showed that the **ODADA** sensor can also be used in practical applications. These findings demonstrate that the new 1,3,4-oxadiazole derivative offers significant potential for developing dual-mode (colorimetric and fluorometric) sensors for the reliable determination of heavy metal ions in environmental and biological samples.

## Graphical abstract

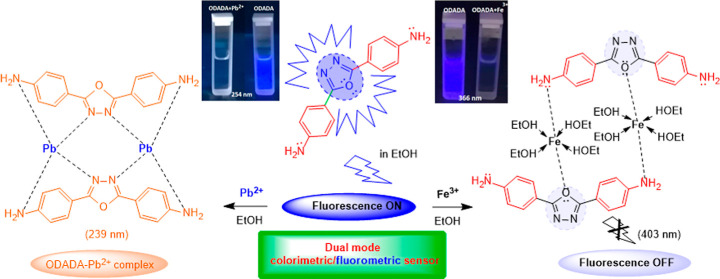

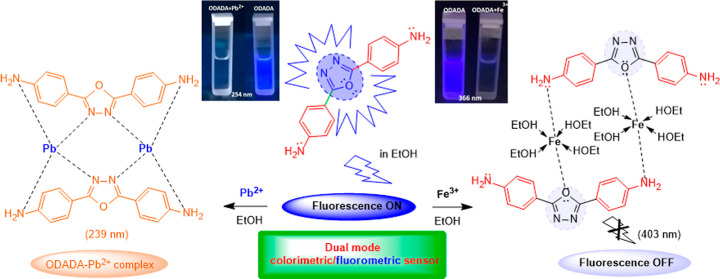

## 1 Introduction

The accumulation of heavy metal ions in environmental and biological systems poses serious threats to human health and ecosystems. Lead (Pb^2+^) ions, in particular, are listed among the most dangerous pollutants by the World Health Organization due to their neurotoxic effects and endocrine-disrupting properties. Iron (Fe^3+^) ions, although an essential element in living metabolism, can lead to oxidative stress and cellular damage when present in excessive amounts. Therefore, the rapid, selective, and sensitive detection of Pb^2+^ and Fe^3+^ ions is of great importance.^1–3^ However, the detection of transition and heavy metal ions such as Pb^2+^ and Fe^3+^ is vital not only in environmental water monitoring but also in industrial organic phase applications, including fuel analysis, petrochemical processes, and organic synthesis quality control. Traditional water-soluble fluorescent probes often fail in these nonaqueous environments due to poor solubility, phase separation, or significant signal interference from high hydration energy. Specifically, in industrial solvents and fuels, trace metal contaminants can cause catalyst poisoning or equipment corrosion, necessitating high-sensitivity analytical tools capable of operating directly in organic media. Therefore, developing probes that function efficiently in organic solvents provides a distinct advantage for real-time monitoring where conventional aqueous assays are inapplicable.^4–6^

Traditional analytical methods (atomic absorption spectroscopy, ICP–MS, etc.) offer high accuracy but require expensive equipment, complex sample preparation, and long analysis times. In recent years, colorimetric and fluorometric approaches have gained prominence, with the aim of developing simple and portable sensor systems. These methods allow for the determination of ions through color changes or fluorescence signals observed with the naked eye.^7,8^

Oxadiazoles are five-membered aromatic heterocyclic compounds containing two nitrogen atoms and one oxygen atom. Different isomers within this class include 1,2,4-oxadiazole, 1,2,3-oxadiazole, 1,3,4-oxadiazole, and 1,2,5-oxadiazole. 1,3,4-Oxadiazole, in particular, is one of the most studied isomers due to its broad range of biological and pharmacological activities (antimicrobial, antibacterial, antifungal, antiviral, antitumor, anticancer, anti-inflammatory, analgesic, etc.) and applications in agricultural chemicals (such as insecticides and herbicides).^9–12^ Furthermore, oxadiazole derivatives have been shown to exhibit Thermally Activated Delayed Fluorescence (TADF) in organic light-emitting diodes (OLEDs) and are widely used in organo-electronics due to their superior electron transport and hole-blocking capabilities. Also, some oxadiazoles exhibit aggregation-induced fluorescence emissions in solid state or nanoparticle forms in bioimaging of some cancer cell lines. Moreover, some of the oxadiazoles function as ester and amide bioisosteres, and compounds bearing an oxadiazole ring have also been successfully evaluated in the selective detection of metal ions or as pH sensors in very acidic conditions.^13–16^

This study investigated the photophysical and electronic properties of 4,4′-(1,3,4-oxadiazole-2,5-diyl)­dianiline (**ODADA**) in solid and solution states and the usability of a new 1,3,4-oxadiazole-based probe **ODADA** as a colorimetric sensor for Pb^2+^ ions and a fluorometric sensor for Fe^3+^ ions. The aim of the study is to contribute to the monitoring of heavy metal ions in environmental and biological settings by providing a low-cost, fast, and selective detection platform.

## 2 Experimental Section

### 2.1 Materials and Equipment

All solvents, including those from Sigma and Merck, as well as quinine sulfate (Fluka) and sulfuric acid (Sigma), were used as received without further purification. Various metal salts, including NaCl, AgCH_3_COO, MgCl_2_·6H_2_O, LiCl, BaCl_2_·2H_2_O, CdCl_2_, ZnCl_2_·H_2_O, CuCl_2_·2H_2_O, CrCl_2,_ NiCl_2_, KCl, MnCl_2_·4H_2_O, HgCl_2_, Pb­(CH_3_COOH)_2_·3H_2_O, CaCl_2_·2H_2_O, FeSO_4_·7H_2_O, CoCl_2_·6H_2_O, CrCl_3_, AlCl_3_ and FeCl_3_ were obtained from Sigma-Aldrich and utilized as metal ion sources. Reactions were monitored on silica gel 60 F_254_ plates and visualized under UV light. The FTIR spectrum was recorded on a Thermo Scientific Nicolet iS20 FT-IR Spectrometer. ^1^H and ^13^C NMR spectra were obtained on a Bruker DPX 400 spectrometer at ambient temperature. Chemical shifts (δ) are given in ppm relative to TMS as downfield; DMSO-*d*_6_ was utilized as the solvent, and the coupling constant (*J*) was reported in hertz (Hz). High-resolution mass spectrometry (HRMS) analysis was done using a Waters LCT Premier XE oa-TOF Mass Spectrometer. The UV–vis absorption and fluorescence spectra were recorded using a Shimadzu UV-1800 spectrophotometer and an Agilent Technologies Cary Eclipse Fluorescence spectrophotometer, respectively.

### 2.2 Synthesis of 4,4′-(1,3,4-Oxadiazole-2,5-diyl)­dianiline (**ODADA**)^17^

To the solution of bis­(4-nitrophenyl)-1,3,4-oxadiazole (0.89 mmol) in acetic acid/water (25 mL + 5 mL/1 mmol), zinc powder (8.9 mmol) was added portionwise at 25 °C. The mixture was stirred for 2 h and filtered, and the solvent was evaporated under reduced pressure. The residue was suspended in distilled water. The aqueous phase was treated with a %20 NaOH solution until pH > 10 was reached, then filtered, washed with water, and dried to get **ODADA**.

#### 2.2.1 Characterization Data

Pale-yellow solid. Yield: 0.200 g (90%), mp 296.5–297.4 °C. *R*_f_ (50% EtOAc/hexane) 0.20. IR (ATR, cm^–1^): *v* = 3307–3200 (NH_2_ str), 2929–2853 (aliph. CH), 1610 (C=N), 1563 (arom. C=C str.), 1494 (C=C str.), 1284, 1176 (C–O), 1013, 838, 800, 747; ^1^H NMR (400 MHz, DMSO-*d*_6_) δ: 7.71 (d, *J* = 8.7 Hz, 4H, arom CH × 4), 6.68 (d, *J* = 8.7 Hz, 4H, arom CH × 4), 5.89 (s, 4H, 2 × NH_2_); ^13^C NMR (DMSO-*d*_6_, 100 MHz, ppm) δ: 163.8 (C=N), 152.4 (C–NH_2_), 128.2 (CH, arom.), 114.0 (CH, arom.), 110.6 (C=C-quart.). HRMS (TOF-MS ESI^+^): M^+^, found 252.10110. C_14_H_12_N_4_O requires 252.10111.

### 2.3 Sensory Applications of **ODADA**

Sensor studies were conducted using **ODADA** stock solutions prepared in ethanol. To obtain fresh probe samples in various media, the solvent was removed via evaporation. All experimental measurements were performed at a constant concentration of 2 μM and maintained at room temperature.

### 2.4 UV–Vis Absorption and Fluorescence Spectroscopy

Quartz cuvettes were used as sample holders for absorption and fluorescence measurements. Steady-state fluorescence measurements were performed using an excitation wavelength of 350 nm, with emission intensities recorded over a range of 360–700 nm. The fluorescence quantum yields for all **ODADA** samples were determined according to previously established protocols. Detailed methodologies regarding these calculations are provided in the Supporting Information.

## 3 Results and Discussion

### 3.1 Synthesis and Characterization of 4,4′-(1,3,4-Oxadiazole-2,5-diyl)­dianiline (**ODADA**)

Synthesis of **ODADA** was achieved via a simple, multistep, and efficient way involving a sequence of condensation, cyclization, and reduction reactions. In the last step, **ODADA** was obtained in good yield by the reduction of 2,5-bis­(4-nitrophenyl)-1,3,4-oxadiazole and further purified by recrystallization (Scheme 1).

**Scheme 1:**
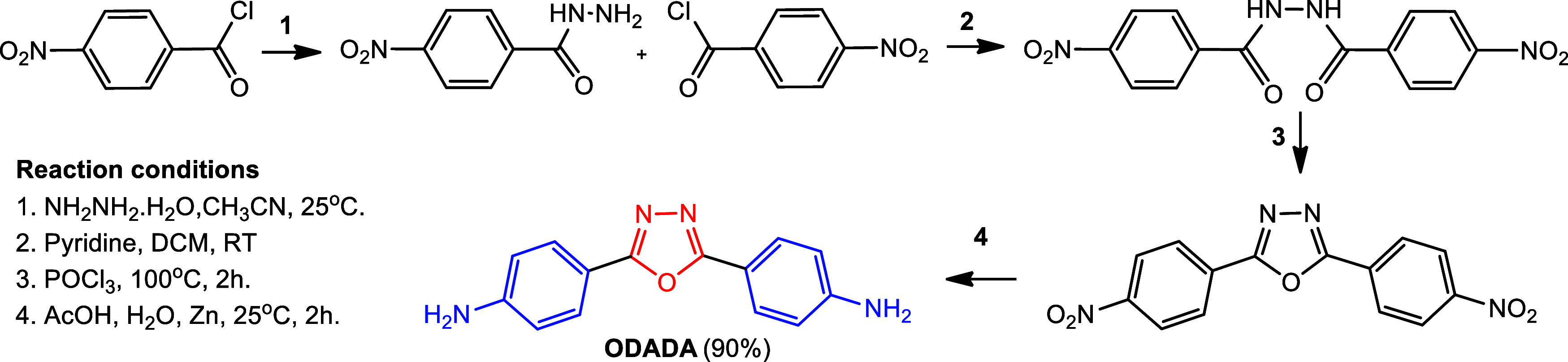
Synthesis of **ODADA**

The structure of **ODADA** was elucidated by FTIR, NMR, and mass analyses. In the FTIR spectrum of **ODADA**, strong amine NH and also C=N and C=C stretching absorption peaks at 3200–3307, 1610, and 1494 cm^–1^, respectively, supported the formation of **ODADA**. Besides, two distinct doublets at 7.7 and 7.0 ppm and a singlet peak at 5.89 ppm in proton NMR proved the presence of aromatic CH and NH_2_ protons in the expected structure of **ODADA**. Moreover, three quaternary carbon peaks resonating at around 110, 152, and 163 ppm in the ^13^C NMR of **ODADA** supported the product formation (Figure 1). Finally, predicted HRMS of **ODADA** was seen in good agreement with its experimental HRMS result (see Figure S1 in Supporting Information).

**Figure 1:**
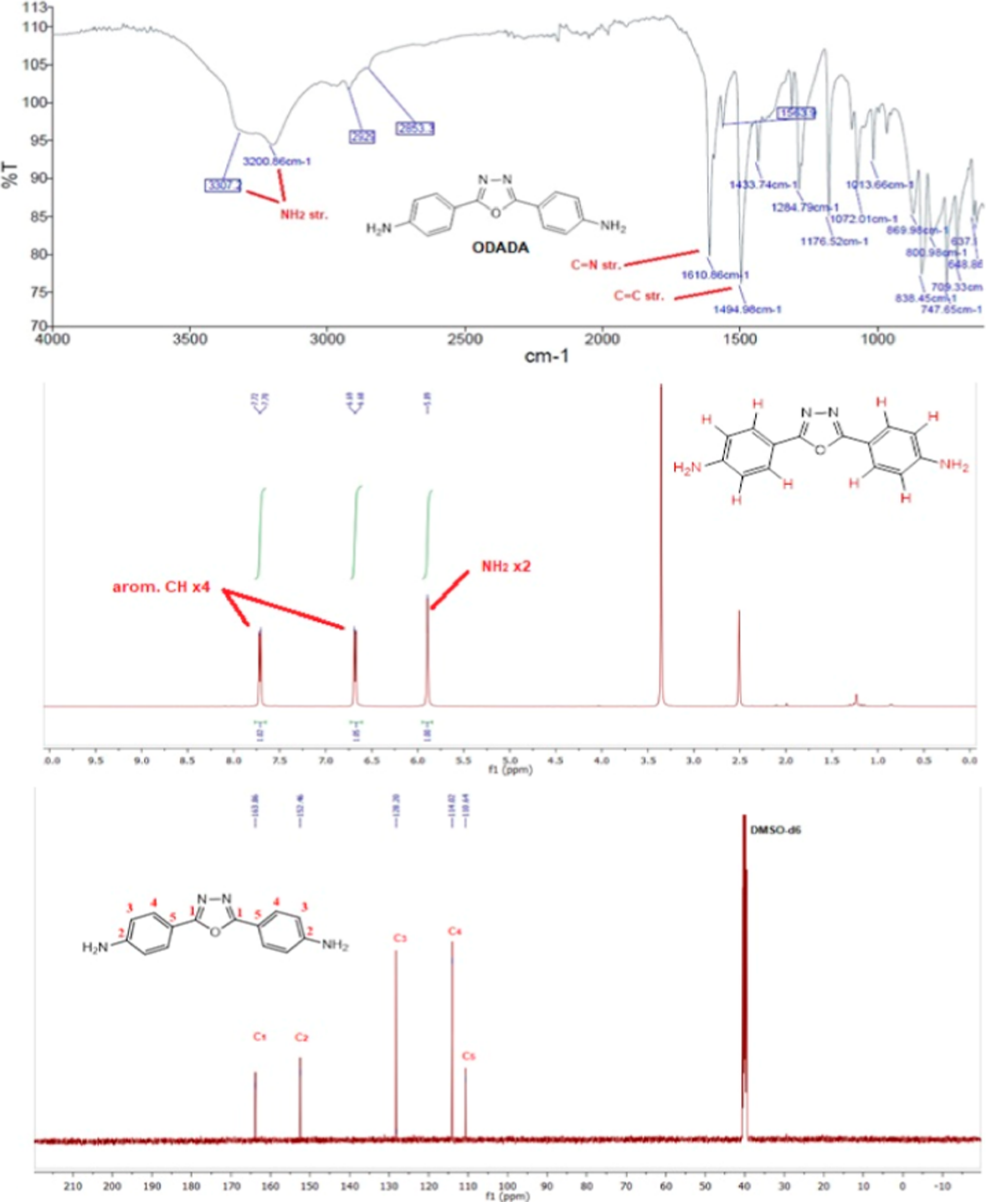
Characterization of **ODADA** with IR and NMR data.

### 3.2 DFT Calculations for Structure Optimization and Electronic Properties of **ODADA**

In order to theoretically elucidate the stable geometry, electronic structure, charge distribution, and potential intramolecular charge transfer (ICT) behavior of the designed and synthesized 1,3,4-oxadiazole-based donor–π–donor (D–π–D) system (**ODADA**), density functional theory (DFT) calculations were performed using Gaussian 09 W software^18^ with the B3LYP/6-311G­(d,p) basis set.^19,20^ All frequencies obtained from the performed full-geometry optimization and vibrational frequency calculations were found to be positive. Figure 2a shows the optimized molecular geometry of the compound **ODADA** in the gas phase, which confirms that the oxadiazole ring exhibits a highly planar conformation with aniline units on either side. This structural arrangement facilitates efficient π-electron delocalization throughout the molecule, indicating that the central unit (i.e., oxadiazole unit) plays an important role in electronic coupling between the terminal donor units. This is also supported by the dihedral angle values calculated across representative torsional axes. The calculated dihedral angles for the ground state (S_0_) are −0.253° and −0.249° for C4–C1–C16–C18 and C5–C2–C6–C8, respectively, and +179.755° and +179.695° for O3–C1–C16–C18 and N5–C2–C6–C7, respectively, clearly demonstrating that **ODADA** has an almost ideal planar geometry. In addition, the electrostatic potential surface (ESP) of **ODADA** was obtained and is depicted in Figure 2b. The ESP map reveals that the electron density (i.e., red regions) is concentrated on the central oxadiazole ring containing electronegative nitrogen and oxygen atoms, as expected, while the end regions containing –NH_2_ groups exhibit a more electropositive character (i.e., blue regions). The obtained asymmetric charge distribution supports the presence of charge separation and permanent polarization within the molecule, consistent with the calculated ground-state dipole moment having a high value of 4.8558 D. This also points to the interaction potential of the structure in polar solvents. On the other hand, the distributions of the Frontier molecular orbitals (FMOs) of **ODADA**, namely, the highest occupied molecular orbital (HOMO) and the lowest unoccupied molecular orbital (LUMO), as well as their energy levels and the energy difference between these levels, have also been computed. As seen in Figure 2c, HOMOs are mostly localized on the donor aniline group, while with excitation, LUMOs redistribute toward the electron-withdrawing oxadiazole ring. This redistributed molecular orbital pattern indicates the presence of a π-bridge-mediated delocalized ICT character within the D–π–D structure, rather than a classical end-to-end charge transfer. These findings are also consistent with the ESP map, which points to more negative potential regions around heteroatoms, and the molecule’s high ground-state dipole moment.

**Figure 2:**
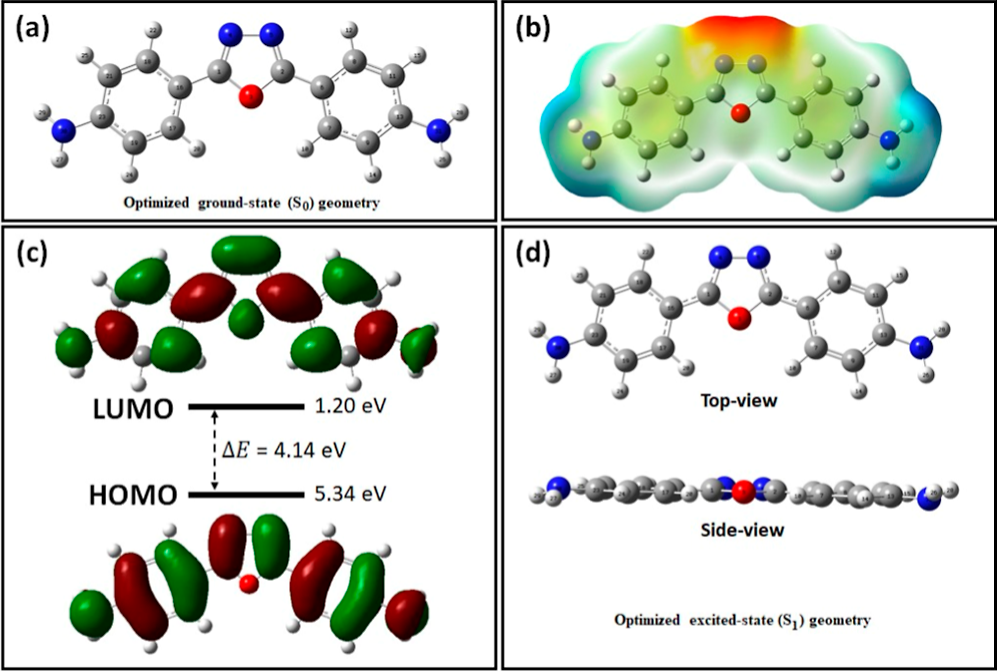
(a) Optimized molecular ground-state (S_0_) geometry, (b) electrostatic potential surface (ESP), and (c) frontier molecular orbital (HOMO and LUMO) distributions of **ODADA**. In the ESP map, the color scale ranging from red to blue represents electron-rich (negative potential) and electron-poor (positive potential) regions, respectively. (d) The top and side views of the optimized excited-state (S_1_) geometry of **ODADA** obtained from TD-DFT calculation.

Additionally, the energy difference between the HOMO and LUMO was calculated to be 4.1375 eV, suggesting a semiconductor-like structure capable of absorbing ultraviolet light. On the other hand, global reactivity descriptors, such as chemical potential (μ), electronegativity (χ), chemical hardness (η), softness (σ), and electrophilicity (ω), were determined using the Frontier molecular orbital energy gap (Δ*E*) to quantitatively evaluate the electronic stability, charge exchange ability, and response to environmental perturbations of **ODADA**. All these parameters are summarized in Table 1.

**Table 1:** Calculated Global Reactivity Descriptors of the Compound **ODADA**: Energies of HOMO and LUMO (*E*_HOMO_ and *E*_LUMO_), Energy Gap between HOMO and LUMO Levels (Δ*E*), Chemical Potential (μ), Electronegativity (χ), Chemical Hardness (η), Softness (σ), and Electrophilicity (ω)

*E*_HOMO_ (eV)	*E*_LUMO_ (eV)	Δ*E* (eV)	μ (eV)	χ (eV)	η (eV)	σ (eV^–1^)	ω (eV)
–5.3424	–1.2049	4.1375	–3.2737	3.2737	2.0688	0.2417	2.5903

In this context, the chemical potential (μ), representing the tendency of an electron to escape from a system, was calculated as −3.2737 eV for the molecule. In addition, the electronegativity (χ) of the molecule was found as 3.2737 eV, directly related to the chemical potential. This reveals that **ODADA** has the capacity to stabilize the electron density coming from the external environment, which presents a picture consistent with the electron-withdrawing tendency of the heteroatom-containing structure. Furthermore, the hardness (η) of the molecule, found as 2.0688 eV, indicates that it has high kinetic stability in the ground state and resistance to sudden electronic charge exchange with its surroundings, consistent with a moderate HOMO–LUMO energy gap (4.1375 eV). In the context of hardness, the value suggests that **ODADA** is not completely inert, but also not an excessively soft and unstable system, and therefore can be polarized in a controlled manner. Besides, the softness (σ) of the molecule, calculated as 0.2417 eV^–1^, indicates that the system maintains a certain level of polarization capacity. These findings point out that **ODADA** maintains its structural integrity and may exhibit measurable changes in its electronic structure under suitable external interactions (e.g., ion binding). On the other hand, the electrophilicity index (ω), which measures a molecule’s ability to accept charge from its environment, was calculated to be 2.5903 eV. This value suggests that **ODADA** has a moderately electrophilic character rather than being a strong electrophile. It also indicates that it possesses a stable electronic structure, allowing it to interact effectively with electron-rich species. All these results, as considered together with the redistributed HOMO–LUMO locations observed in the FMO analysis and the significant electrostatic potential differences revealed in the ESP map, suggest that **ODADA** may be a system capable of responding to an external stimulus (e.g., metal cation or anion binding) via intramolecular charge redistribution. In particular, the symmetric D–π–D structure of **ODADA**, formed by the acceptor nature of the oxadiazole ring at the center and the donor character of the amine groups at the ends, may allow for the alteration of this delicate reactivity balance (e.g., signal generation due to hardness–softness change) upon binding of target ions. This may lead to colorimetric and/or fluorometric responses to external stimuli, such as metal ions.

Furthermore, it is known in the literature that significant geometric rearrangements can occur in the excited state in donor–acceptor conjugated systems, and twisted charge transfer (TICT) states, characterized by torsional bending between donor and acceptor groups, can occur. Such a structural change can significantly affect the excited state electronic structure and consequently the photophysical properties. Therefore, in order to accurately reveal the excited state behavior of **ODADA** and to directly understand the possible TICT formation, geometry optimization was performed for the lowest energy singlet excited state (S_1_) using time-dependent density functional theory (TD-DFT). The results reveal that **ODADA** maintains a highly planar conformation in the excited state, similar to its ground state (see Figure 2d). The fact that the dihedral angles calculated across representative torsional axes between the aniline (donor) and oxadiazole (acceptor) groups remain around ∼0° and ∼180° (e.g., −0.180° and −0.056° for C4–C1–C16–C18 and C5–C2–C6–C8, respectively, −179.946° and −179.817° for O3–C1–C16–C18 and N5–C2–C6–C7, respectively) clearly shows that the molecule does not undergo significant torsional rearrangement in the excited state. The same dihedral angles in the ground state reveal that the postexcitation structural change is negligible and that the π-conjugated planar structure of the molecular skeleton is preserved. In other words, it does not find any significant rotation (∼90°) required for the formation of the TICT mechanism in donor–acceptor systems in the **ODADA**; instead, an electronic structure that is stabilized by π-delocalization, while maintaining a planar geometry, is present. This indicates that the excited state properties of the system are governed by charge redistribution (i.e., ICT) along the conjugated skeleton rather than by torsional distortion (i.e., TICT). In addition, the excited-state dipole moment was found to be 5.0185 D with a TD-DFT single-point calculation on the optimized S_1_ geometry. Compared with the ground state, this limited increase in the dipole moment after excitation suggests increased charge separation in the structure. However, the absence of the large dipole increase expected in a strong TICT case also supports the conclusion that **ODADA** exhibits planar and delocalized ICT characteristics.

### 3.3 Photophysical Studies

#### 3.3.1 Solid-State Optical Behavior of the Probe **ODADA**

The photophysical properties of **ODADA** with the D–π–D structure were first studied in its solid phase using steady-state photoluminescence (PL) spectroscopy. The measurements were conducted at room temperature with an excitation wavelength of 350 nm. Figure 3a shows the PL emission curves of the compound **ODADA** for its three different solid-state cases, such as pristine powder (i.e., as-synthesized powder), in a KBr matrix, and drop-cast solid film on a glass substrate. The collected solid-state PL spectra reveal that **ODADA** exhibits an emission behavior that is strongly dependent on sample preparation conditions. As seen in Figure 3a,b, the main PL emission peak shifts between the 511–543 nm range, depending on the sample type, with a shoulder appearing constantly around 425 nm. Such PL emission profiles with dual bands are often reported in the literature as typical behavior, indicating the simultaneous presence of locally excited (LE) and environmentally responsive intramolecular charge transfer (ICT) contributions.^21–24^ It was found that the PL emission maximum appears at 511 nm with a quite large full-width half-maximum (*fwhm*) of 237.6 nm for the pristine powder, suggesting that different molecular packing motifs and aggregation types contribute simultaneously. These types of asymmetric PL emissions with broad bandwidth are frequently observed in powder form, where inhomogeneous broadening predominates, especially in molecules with ICT characteristics and high dipole moments.^25,26^ On the other hand, in the case of the film sample prepared by drop-casting on glass, it is noteworthy that as the main PL emission maximum shifts to 528 nm, the bandwidth narrows dramatically, dropping to *fwhm* ∼51.6 nm. This behavior can be explained by the fact that in film form, the molecules are situated in a more uniform microenvironment, allowing a single dominant emissive species to stand out. It is known that when conjugated and ICT molecules are more regularly packed in film form, or when a particular aggregation geometry becomes dominant, the emission bands narrow and spectral homogeneity increases.^27–30^ In addition, the red-shifted PL profile observed in the drop-casting film indicates that the ICT mechanism can be predominantly responsible for this narrower emission band. Furthermore, due to the rough solid film preparation condition (i.e., drop-casting), the broad shoulder at 425 nm still emerges with lower intensity in the emission profile. In the case of the ground sample within the KBr matrix, the main PL emission maximum is further shifted to the longer wavelengths, i.e., 543 nm, and the subsequent increase in the bandwidth to 205.2 nm. This indicates that an ionic matrix with a high electrostatic field strongly stabilizes the excited state. It has been previously reported that ionic matrices such as KBr have a significant stabilizing effect on excited states, especially those with polar and ICT characteristics, and consequently, the emission shifts to longer wavelengths.^30,31^ Besides, the grinding process and change of dielectric medium surrounding the compound can be responsible for the increase in bandwidth.^31^ On the other hand, observing the shoulder band in the PL spectra that remains constant at approximately 425 nm in all solid samples suggests that this emission contribution is largely insensitive to sample preparation conditions and is more likely related to an emission mechanism based on a locally excited (LE) state that is relatively less influenced by environmental factors compared to the ICT state. Moreover, the significant change in the relative intensity of this shoulder at 425 nm compared to the main band among the powder, film, and KBr samples indicates that the balance between LE and ICT contributions to the emission is readjusted depending on the microenvironment (see Figure 3c). Additionally, according to the International Commission on Illumination (i.e., CIE 1931), color coordinates of **ODADA** were determined for various solid-state sample preparation methods. Figure 3d depicts the CIE 1931 color coordinates for the compound in various solid forms, revealing that the emission color changes significantly depending on the sample preparation conditions. The position obtained for the pristine powder sample (*x* = 0.3404, *y* = 0.3866), corresponding to CCT = 5250 K, indicates a near-neutral to warm white emission; however, the sample prepared in the KBr matrix (*x* = 0.2820, *y* = 0.3432, CCT = 8043 K) exhibits a cool white (i.e., blue-shifted) emission with a higher color temperature. This shift is consistent with the ionic and highly polar microenvironment of KBr, which differentially stabilizes the ICT-weighted excited state and redistributes spectral weight toward shorter wavelengths. For drop-casting film, the color coordinate (*x* = 0.2614, *y* = 0.5056) of **ODADA** shows a significant shift toward the green region and is not defined by a single CCT value. Overall, these results demonstrate that the color coordinates and perceived color temperature of the same compound can be tuned under different solid-state preparation conditions, thus indicating that the emission color can be effectively controlled by morphology and microenvironment.

**Figure 3:**
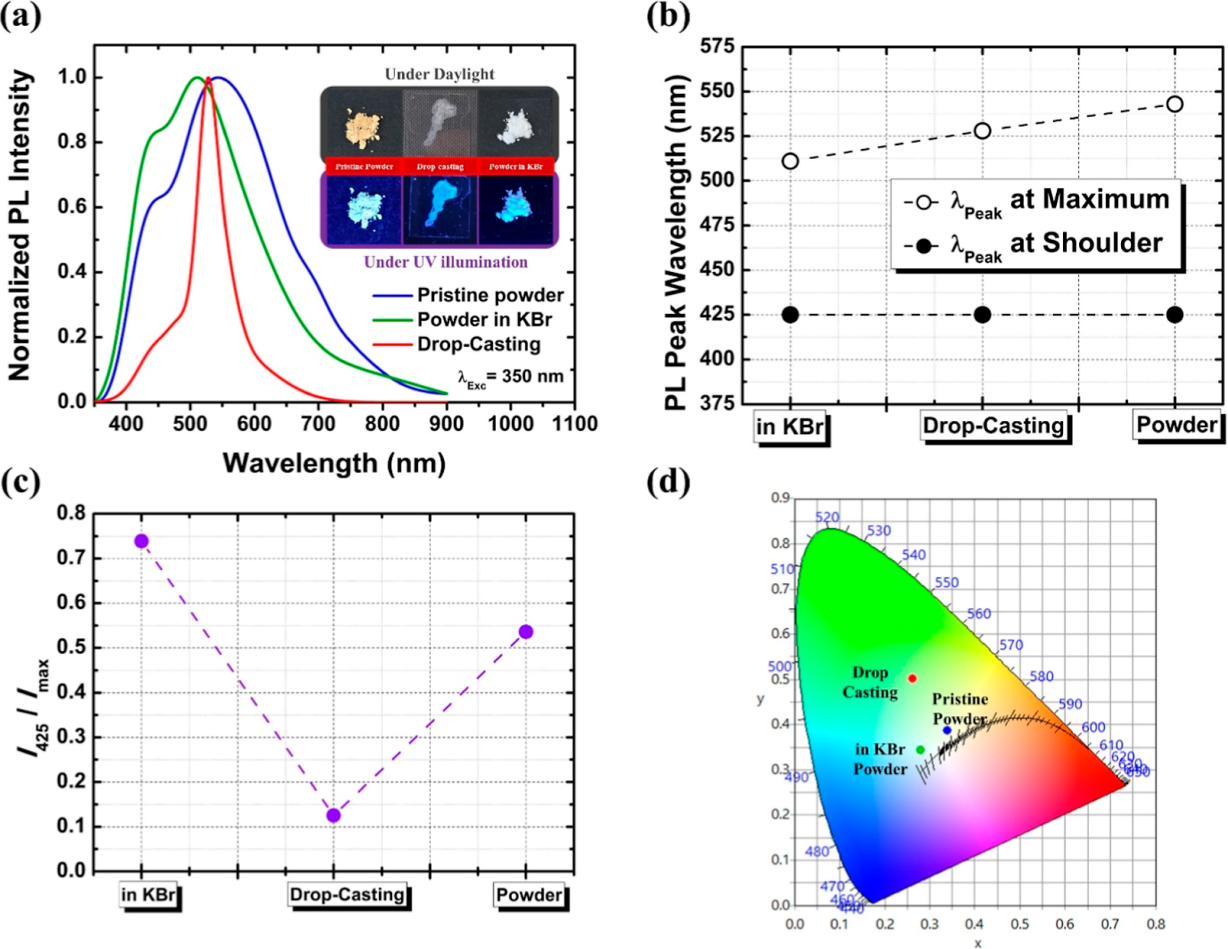
(a) Normalized solid-state PL spectra for pristine powder, powder ground in the KBr matrix, and drop-casting film onto glass under 350 nm excitation. The inset of (a) shows the photos of the samples under daylight and UV illumination. (b) Evolution of the PL peak position of the main emission and shoulder band for solid samples of the compound. (c) The ratio of the relative emission intensity of the shoulder band to the main emission band. (d) The color coordinates of the samples according to the CIE 1931.

#### 3.3.2 Solvent Effect on the Optical Behavior of **ODADA**

The UV–vis absorption and fluorescence spectra of **ODADA** were recorded in 13 different solvents such as toluene, ethyl acetate, diethyl ether, dichloromethane (DCM), tetrahydrofuran (THF), chloroform, dimethylformamide (DMF), dimethyl sulfoxide (DMSO), acetonitrile (ACN), isopropanol (i-PrOH), methanol (MeOH), ethanol (EtOH), and water at room temperature. The probe **ODADA** presented absorption peaks in the range of 250–300 nm and 300–400 nm in almost all selected solvents (Figure S2a). The UV–vis spectral analyses conducted in various solvents revealed two distinct absorption regions, corresponding to specific electronic transitions within the molecule. The absorption bands in the 300–400 nm range are attributed to π–π* transitions with significant Intramolecular Charge Transfer (ICT) character, as supported by DFT calculations showing HOMO localization on aniline donors and LUMO redistribution toward the oxadiazole core. The higher-energy features observed between 250 and 300 nm are assigned to localized π–π* transitions within the aromatic subunits and the oxadiazole ring. While n−π* contributions from the heteroatoms are possible in this region, the high oscillator strengths of the π–π* transitions dominate the electronic profile, consistent with the calculated planar geometry and efficient electronic coupling. These observations highlight the influence of the molecular framework on the electronic transitions and provide insight into the photophysical behavior of the compound across different solvent environments.^32^

Fluorescence measurements were also taken at an excitation wavelength of 350 nm for the probe **ODADA** (Figure S2b). Since the oxadiazole structure contains aromatic structures that are sensitive to n−π* and π–π* transitions, the fluorescence properties of these compounds are significantly affected by solvent–solute interactions.^33,34^ It is determined that the probe **ODADA** shows fluorescence properties in the range of approximately 380–470 nm. It was observed that fluorescence maxima were at shorter wavelengths in low-polarity solvents such as toluene, diethyl ether, and THF, while emission maxima were at longer wavelengths in high-polarity solvents such as DMSO and DMF. Due to both the high polarity of alcohol species and their ability to form hydrogen bonds, a significant red shift was observed in the fluorescence band maxima of **ODADA** when the solvent was alcohol. Fluorescence quantum yields of the probe **ODADA** in all studied solvents were also calculated. It was determined that the probe **ODADA** had moderate fluorescence quantum yields, especially in nonpolar and polar aprotic (except for DMSO) solvents (Table 2). As the polarity of the solvent increased, a noticeable reduction in quantum yield was recorded, presumably resulting from inadequate dielectric effects or the lack of effective π–π stacking in the hydrogen bonding environment.^35^

**Table 2:** Spectroscopic and Photophysical Parameters of **ODADA** in Different Solvents

compound	solvent	λ_abs_ (nm)	ε × 10^4^ (M^–1^ cm^–1^)	λ_em_ (nm)	Φ
**ODADA**	toluene	321	2.45	383	0.34
	diethyl ether	325	2.90	383	0.27
	THF	330	2.51	390	0.30
	ethyl acetate	276/324	2.63	386	0.36
	chloroform	261/327	3.49	392	0.14
	DCM	262/323	2.66	385	0.42
	DMF	337	2.57	401	0.13
	DMSO	339	2.45	405	0.01
	ACN	326	2.42	394	0.27
	i-PrOH	335	2.45	403	0.14
	EtOH	336	2.50	403	0.12
	MeOH	334	2.81	403	0.16
	water	323	2.40	468	0.02

### 3.4 Sensory Applications of **ODADA** toward Different Metal Ions

The interaction capacity of the probe (**ODADA**) with metal ions was evaluated using steady-state fluorescence spectroscopy and UV–vis. absorption techniques in ethanol. For this purpose, 10 μM concentrations of K^+^, Li^+^, Ag^+^, Na^+^, Mg^2+^, Ba^2+^, Cd^2+^, Ca^2+^, Zn^2+^, Mn^2+^, Ni^2+^, Hg^2+^, Cu^2+^, Cr^2+^, Co^2+^, Fe^2+^, Pb^2+^, Fe^3+^, Al^3+^, and Cr^3+^ ions, prepared by dilution from stock solutions, were used. Each metal ion was added separately to a 5 mL solution containing 2 μM probe **ODADA**, and absorption and fluorescence spectra were recorded for each ion. A new peak was observed at approximately 239 nm in the absorption spectrum of the probe **ODADA** in the presence of Pb^2+^ ions, while no changes were observed in the presence of the other ions studied (Figure 4a). The fluorescence measurement results showed that a quenching in the fluorescence intensity of the probe **ODADA** occurred only in the presence of Fe^3+^ ions via a possible photoelectron transfer (PET) mechanism of 1,3,4-oxadiazole core toward Fe^3+^ ions (Figure 4b). Furthermore, the quantum yield of the probe **ODADA** was calculated in the presence and absence of the studied metal ions (relative to eq S1), and it was determined that the quantum yield of the probe **ODADA** (Φ = 0.12) decreased 4-fold only in the presence of Fe^3+^ ions (Φ = 0.03) (Table 3). These results suggest that the newly synthesized probe **ODADA** can be a colorimetric sensor for Pb^2+^ and a fluorometric sensor for Fe^3+^ ions.

**Figure 4:**
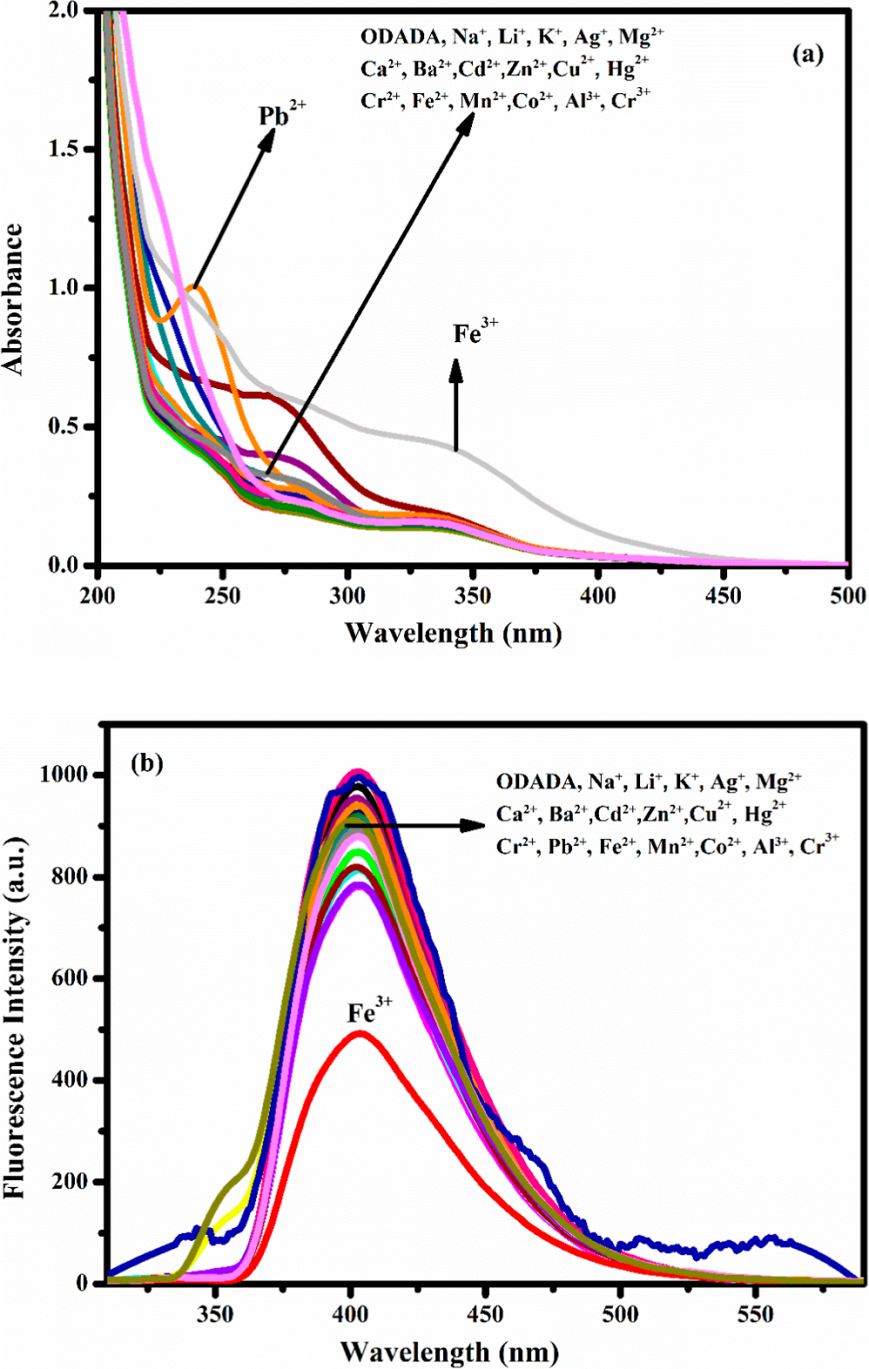
(a) UV–visible absorption and (b) fluorescence spectra of **ODADA** (2 μM) were recorded in ethanol, both in the absence and presence of metal ions (10 μM), with an excitation wavelength of 350 nm.

**Table 3:** Fluorescence Quantum Yields of **ODADA** in the Absence and Presence of the Studied Metal Ions

media	Φ
-	0.12
Na^+^	0.13
Li^+^	0.11
K^+^	0.11
Ag^+^	0.10
Mg^2+^	0.09
Ca^2+^	0.12
Ba^2+^	0.10
Cd^2+^	0.11
Zn^2+^	0.09
Cu^2+^	0.06
Ni^2+^	0.11
Hg^2+^	0.09
Cr^2+^	0.10
Pb^2+^	0.10
Fe^2+^	0.08
Mn^2+^	0.12
Co^2+^	0.13
Fe^3+^	0.03
Al^3+^	0.09
Cr^3+^	0.12

Titration experiments were conducted at different metal ion concentrations to determine the sensitivity of the probe **ODADA** to Pb^2+^ and Fe^3+^ ions. Absorption measurements were performed for the probe **ODADA** in the presence of Pb^2+^ ions in the concentration range of 0–150 μM. As shown in Figure 5a, the intensity of the absorbance peak of the probe **ODADA** at 239 nm increases with increasing concentration of Pb^2+^ ions. The relationship between the concentration of Pb^2+^ ions and the absorbance peak of the probe **ODADA** was determined to be highly linear (Figure 5b). The decrease of the fluorescence peak of the probe **ODADA** at 403 nm with respect to Fe^3+^ ion concentration (0–120 μM) was also investigated, and the concentration–intensity relationship was found to be quite linear (Figure S3a,b). Using all these results, the detection limit (LOD) values of the probe **ODADA** for Pb^2+^ and Fe^3+^ ions were calculated as 0.06 and 0.10 μM, respectively. The fact that the calculated LOD values are quite low compared to studies in the literature shows that the probe **ODADA** is a very sensitive sensor.^36–41^

**Figure 5:**
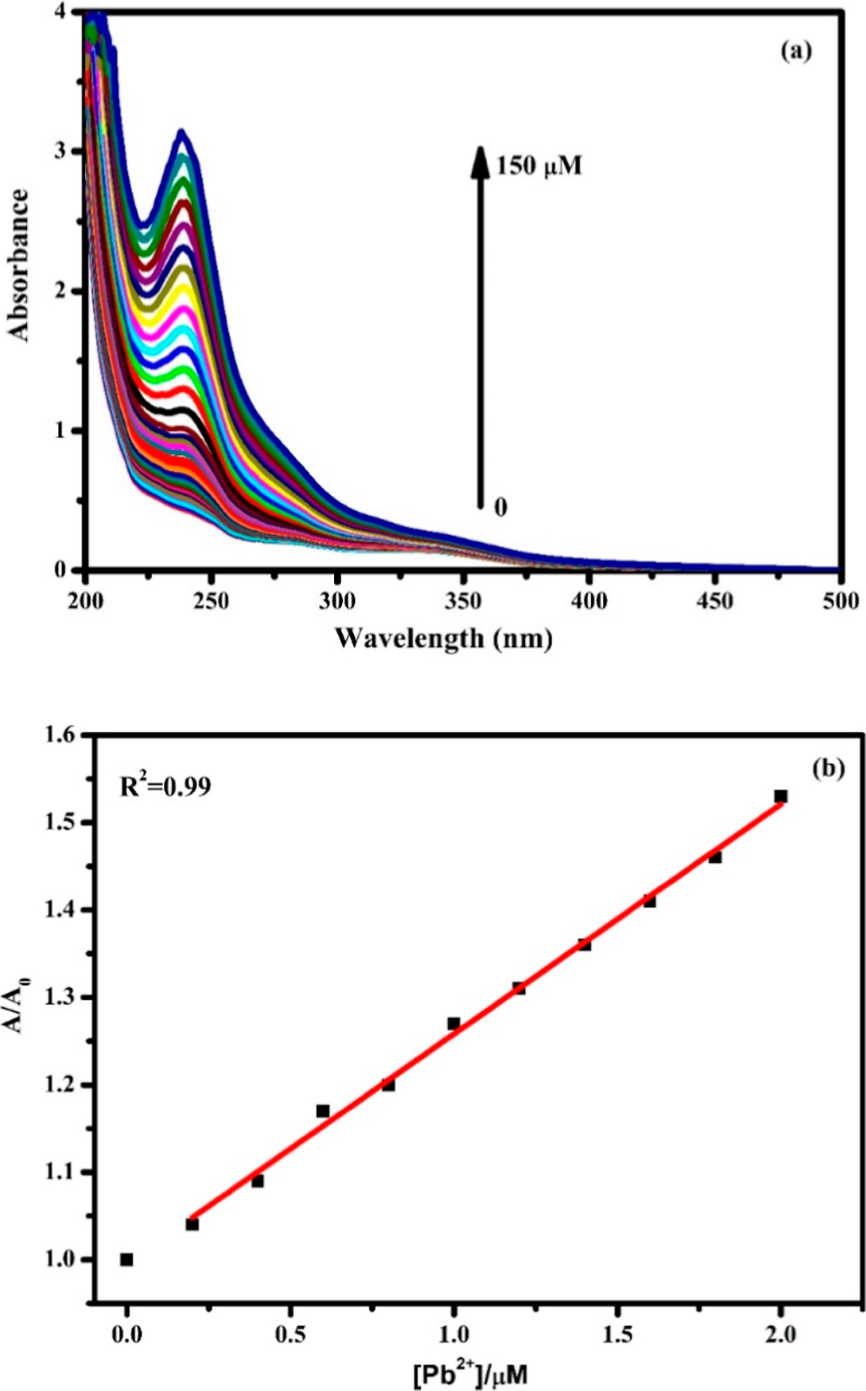
(a) Absorption spectra and (b) variation in the absorption intensity of the probe **ODADA** (2 μM) with increasing Pb^2+^ ion concentration in ethanol.

The stoichiometry of the probe **ODADA** binding to the detected ions (Pb^2+^ and Fe^3+^) was determined using Job’s plot analysis (eq S2). Job’s plots were generated from the absorbance studies of the probe **ODADA** with Pb^2+^ and the fluorescence studies with Fe^3+^. Both graphs show a minimum at approximately 0.5 mole fraction, which reflects that the probe **ODADA** forms a complex with both Pb^2+^ and Fe^3+^ in a 1:1 ratio or 2:2 ratio (Figures 6a and S4a). The association constants (*K*_a_) were also calculated using the Benesi–Hildebrand equation with this determined binding stoichiometry (eq S3). The graphs between 1/*A* – *A*_0_ and 1/[Pb^2+^] and 1/F_0_–F and 1/[Fe^3+^] were determined to be highly linear, and using these graphs, the K_a_ values were calculated as 1.86 and 2.23 M^–1^ for Pb^2+^ and Fe^3+^, respectively (Figure 6b and S4b).

**Figure 6:**
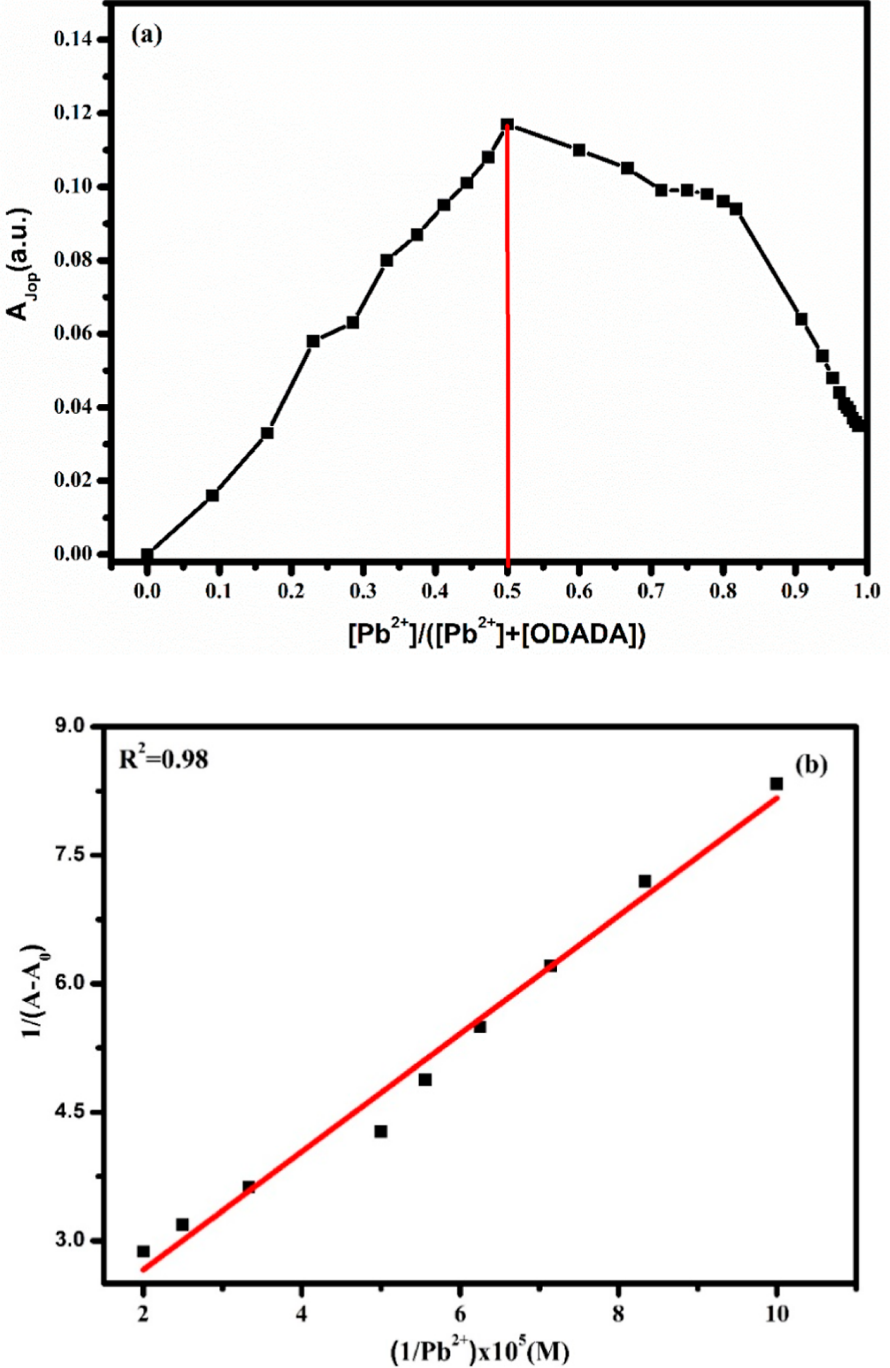
(a) Job’s plot of the probe **ODADA** with Pb^2+^. (b) Benesi–Hildebrand plot based on a 1:1 association stoichiometry between Pb^2+^ and the probe **ODADA** in ethanol.

#### 3.4.1 Dual-Channel Interference Analysis for Pb^2+^ and Fe^3+^

The selectivity of the new sensor **ODADA** for the detected ions was also investigated. Absorption spectra were obtained separately for each of the other ions studied in the presence of Pb^2+^, and the absorbance peak at 239 nm of the probe **ODADA**, resulting from the presence of Pb^2+^ ions, was observed even in the presence of all other ions in the environment (Figure 7a). In addition to the other ions being studied, Fe^3+^ was added to each sample, and the fluorescence spectra of each sample were also analyzed (Figure S5). It was observed that none of the ions studied affected the quenching of the probe **ODADA**’s fluorescence in the presence of Fe^3+^ ions (Figure 7b). These results confirmed that Pb^2+^ and Fe^3+^ ions were detected by the probe **ODADA** even in the presence of other ions being studied in the environment, meaning that none of the other ions being studied interfered.

**Figure 7:**
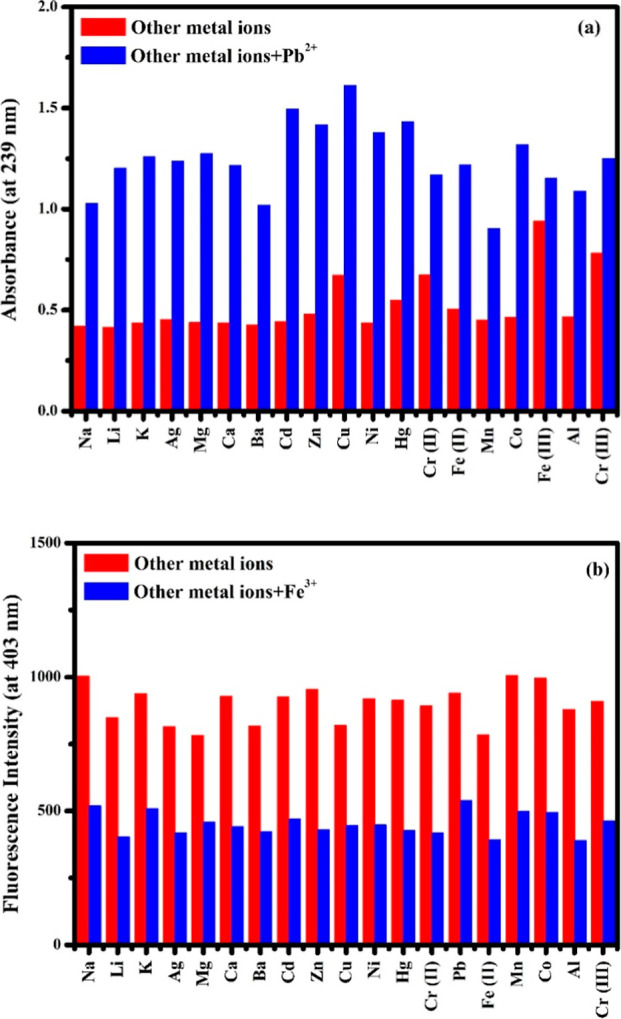
Selectivity profiles of (a) Pb^2+^ and (b) Fe^3+^ ions for the probe **ODADA** in the presence of various metal ions in ethanol.

#### 3.4.2 Response Time and pH Behavior of the Sensor ODADA

The response times of the probe **ODADA** as a sensor for Pb^2+^ and Fe^3+^ ions were also tested. The absorption peak of the probe **ODADA** was recorded between 0.5 and 10 min in the presence of Pb^2+^ ions. The absorption peak of the probe **ODADA** at 239 nm in the presence of Pb^2+^ ions was observed in the first 0.5 min (Figure 8a). Fluorescence measurements of the probe **ODADA** were also taken in the presence of Fe^3+^ ions over the aforementioned time period. The quenching in the fluorescence intensity of the probe **ODADA** in the presence of Fe^3+^ ions occurred within the first 0.5 min (Figure 8b). These data have demonstrated that this new sensor, **ODADA**, an oxadiazole derivative, possesses high sensitivity and selectivity as well as a very fast response time.

**Figure 8:**
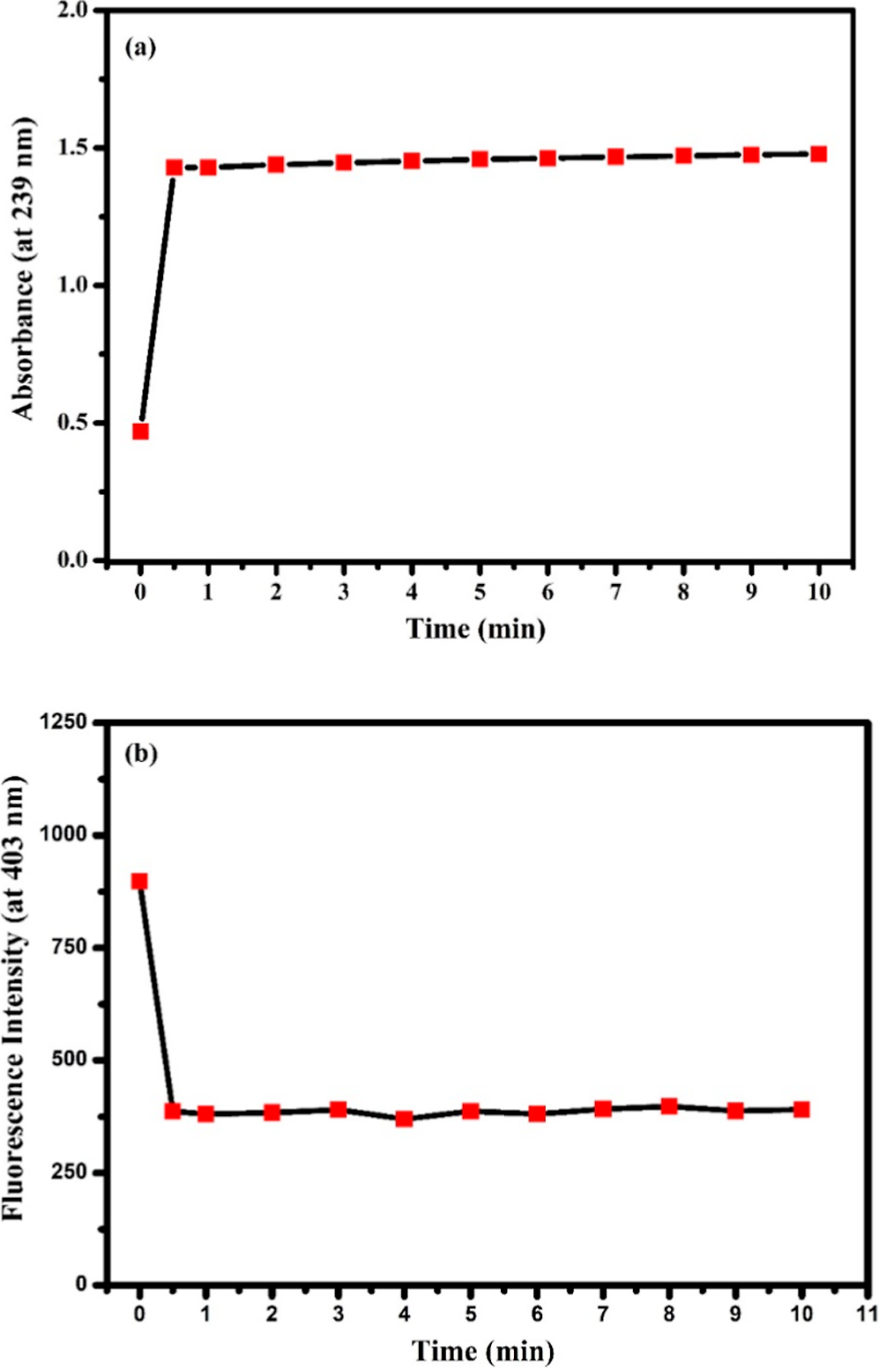
(a) Absorbance enhancement profile of the probe **ODADA** (2 μM) in ethanol upon the addition of Pb^2+^ (10 μM) and (b) fluorescence enhancement profile of the probe **ODADA** (2 μM) in ethanol upon the addition of Fe^3+^ (10 μM) over a time interval of 1 to 10 min.

It is also very important to examine the behavior of sensor systems at different pH values. Therefore, the effect of pH on the absorption and fluorescence properties of **ODADA** was also investigated. The probe exhibited a stable and strong absorption band in the acidic to near-neutral range (pH 2.0 to 6.0). However, at pH 7.0 and above, a significant loss in characteristic absorption intensity was observed. This behavior indicated that **ODADA** is highly susceptible to deprotonation or structural changes in alkaline environments (Figure S6a). The fluorescence intensity of **ODADA** in aqueous buffered systems is extremely low at all pH values due to solvent-induced quenching and competitive protonation of aniline moieties. Although a marginal increase was observed at pH 7.0, the overall quantum yield remains insufficient for reliable metal ion detection in water (Figure S7a). It was observed that the behavior of **ODADA** in pH environments in the presence of Pb^2+^ and Fe^3+^ ions was similar to that in the absence of these ions (Figures S6b and S7b). The lack of selectivity of **ODADA** for the detected ions depending on pH demonstrated that metal ion coordination and fluorescence response were independent of ambient pH.

#### 3.4.3 Sensing Mechanism

FTIR analysis performed to gain insight into the binding interactions of the detected cations (Pb^2+^ and Fe^3+^) with the **ODADA** sensor revealed changes in functional groups at the molecular level. The binding mechanism was predicted by observing changes (peak shifts, intensity changes or disappearance of peaks) at specific wavelengths as the target ion bound to the sensor surface in comparison to recent sensor studies^42–44^ When the FTIR spectrum of **ODADA** was examined, the vibrational bands belonging to the NH_2_, C=N, C=C, and C–O–C groups were observed at 3325 and 3197, 1611, 1442, and 1072 cm^–1^, respectively (Figure 9a). However, in the FTIR spectrum of the **ODADA-Pb** complex, upon complexation, the C=N stretching peak of the 1,3,4-oxadiazole ring shifted to a lower wavenumber (1522 cm^–1^) due to the donation of electrons from the nitrogen to the Pb­(II) metal ion. Besides, the peaks observed near 1330 and 1374 cm^–1^ corresponded to the C–N and C–O–C stretching of the ring, respectively. And the peak at 755 cm^–1^ is associated typically with the out-of-plane wagging vibration of the NH_2_ group due to coordination. Moreover, the disappearance of NH_2_ stretching peaks entirely in the FTIR spectrum of the complex signifies that amine groups are actively involved in the complexation of the lead ion (Figure 9b). In the FTIR spectrum of **ODADA**-Fe^3+^, there was a slight shift in C=N stretching vibrations (1598 cm^–1^), which shows ferric ions were not interacting with the oxadiazole core during complex formation. So, the binding is most likely through oxadiazole C–O–C and –NH_2_ groups of the aniline moiety in a 2:2 stoichiometry. Moreover, the increased intensities of the aliphatic C–H and C–O–C stretching bands clearly indicate that ferric ions form a complex via –OH groups of ethanol (Figure 9c). The interaction mechanisms inferred from these observations are presented in Figure 10.

**Figure 9:**
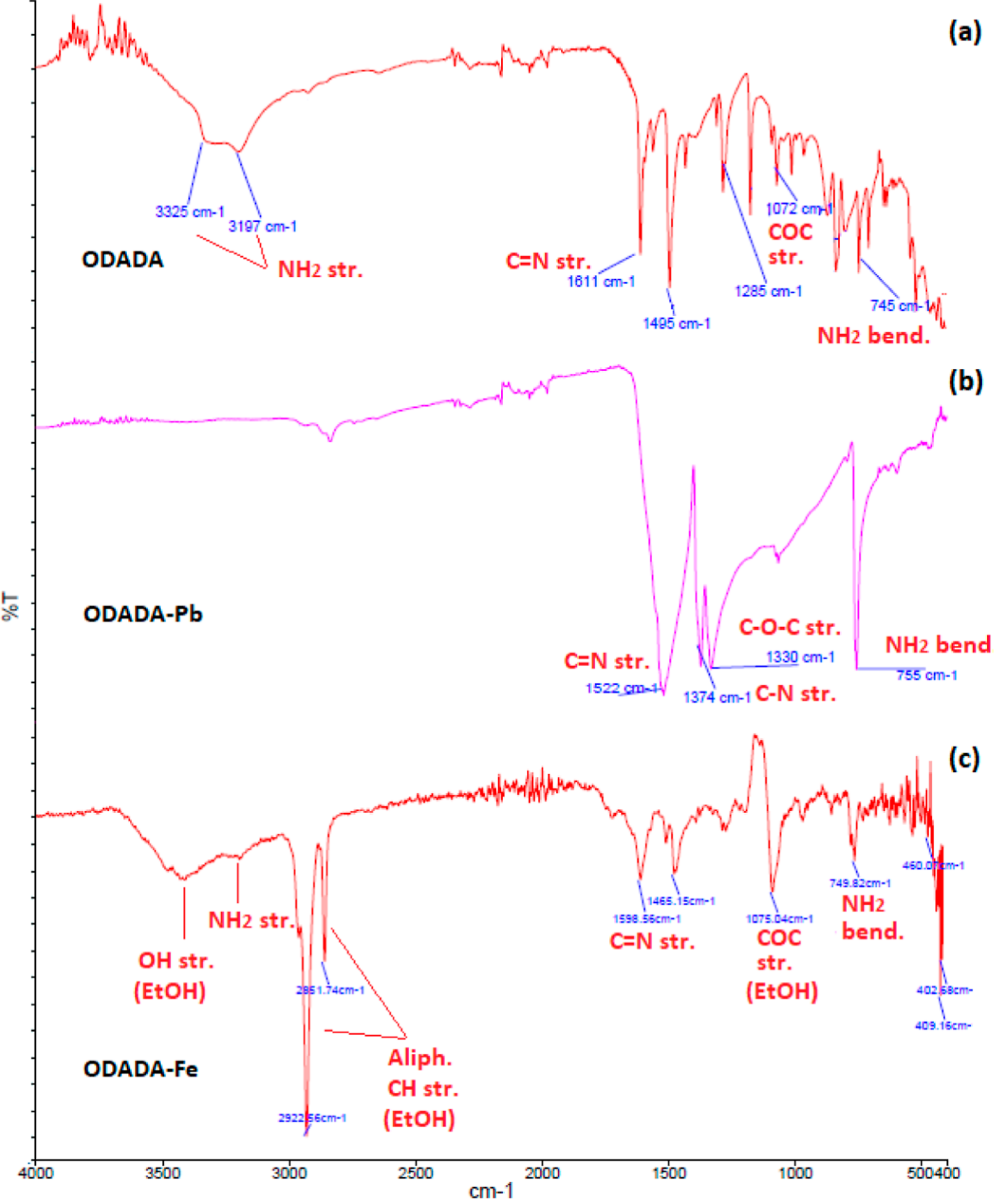
FTIR spectra of (a) free **ODADA**, (b) **ODADA**–Pb^2+^, and (c) **ODADA**–Fe^3+^.

**Figure 10:**
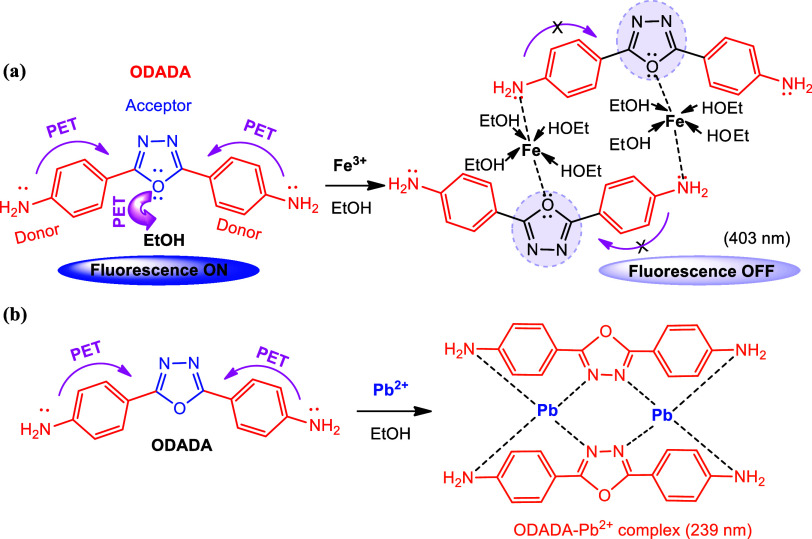
(a) Proposed fluorescence quenching mechanism of Fe^3+^ with **ODADA** in ethanol and (b) proposed binding mechanism of Pb^2+^ with **ODADA** in ethanol.

#### 3.4.4 Comparison with Reported Pb^2+^ and Fe^3+^ Sensors

To evaluate the sensing performance of the proposed probe, a comparative study was conducted with the previously reported Pb^2+^ and Fe^3+^ sensors (Table 4). This table compares our probe to previously reported Pb^2+^ and Fe^3+^ sensors in terms of the limit of detection (LOD), response time, solvent system, and binding constant. As shown in the table, our probe offers a significantly lower limit of detection compared with other studies in the literature, demonstrating practical superiority over existing methods.

**Table 4:** Comparison of Some Selective Chemosensors

solvent system	binding constant (M^–1^)	detection limit (μM)	response time (min)	sensing ions	ref
ethanol	-	1.9	-	Fe^3+^	^6^
ethanol	3.35 × 10^9^	7.48	2	Pb^2+^	^45^
HEPES buffer at 7.4 pH in DMSO solution	-	0.095	-	Fe^2+^ and Pb^2+^	^46^
Tris–HCl buffer	-	13.8		Pb^2+^	^47^
H_2_O	-	0.48	-	Fe^3+^	^48^
THF/H_2_O (7:3, v/v)	1.17 × 10^4^	36.64	-	Cu^2+^, Fe^2+^ and Fe^3+^	^49^
ethanol	0.81 × 10^6^	0.2687	-	Fe^3+^	^50^
gallic and DMF	-	715	-	Pb^2+^	^51^
ethanol	1.86 for Pb^2+^ 2.23 for Fe^3+^	0.06 for Pb^2+^ 0.10 for Fe^3+^	0.5	Pb^2+^ and Fe^3+^	**ODADA** (this work)

#### 3.4.5 Real Sample Tests

A commercial hair dye sample was used to determine the application area of the sensor. A 0.5 g sample of commercial hair dye was dispersed in 50 mL of ethanol and subjected to ultrasonic treatment for 25 min. The mixture was centrifuged at 8000 rpm for 10 min and filtered through a 0.22 μm membrane filter. This prepared matrix was then used for recovery studies by spiking known concentrations of Pb^2+^ and Fe^3+^. Pb^2+^ and Fe^3+^ions were added separately to the prepared hair dye samples at concentrations of 20, 60, and 100 μM. It was performed absorption measurements for the prepared Pb^2+^ samples, and fluorescence measurements for the Fe^3+^ samples. As summarized in Table 5, the recovery values for the detected ions ranged from 94.3% to 139.7%, with a relative standard deviation (RSD) of less than 25%. These results clearly demonstrate that the probe **ODADA** can effectively detect Pb^2+^ and Fe^3+^ ions in real-world consumer products with high accuracy and minimal interference from the complex hair dye matrix.

**Table 5:** **ODADA** for Detection of Pb^2+^ and Fe^3+^ in Real Samples (*n* = 3)

ion	spiked (μM)	found (μM)	recovery (%)	RSD (%)
Pb^2+^	20	27.9	139.7	24.6
	60	66.0	110.0	17.9
	100	94.6	94.6	5.4
Fe^3+^	20	18.9	94.3	8.2
	60	57.2	95.4	9.7
	100	99.7	99.7	0.6

## 4 Conclusions

In conclusion, we have designed and synthesized a new oxadiazole aniline derivative (**ODADA**) in good yield through a multistep, efficient synthetic route. The structure of **ODADA** was confirmed by FTIR, ^1^H NMR, ^13^C NMR, and HRMS analyses. It was found that **ODADA** exhibits environment-sensitive photophysical properties both in the solid and solution phases. Also, this study demonstrated **ODADA** can be used as a dual-mode sensor for the selective determination of Pb^2+^ and Fe^3+^ ions. **ODADA** provides rapid detection by exhibiting a significant colorimetric response toward Pb^2+^ ions, while its strong fluorometric response toward Fe^3+^ ions ensures high sensitivity. In the analyses performed, the limit of detection (LOD) for Pb^2+^ was determined as 0.06 μM, and the linear range was 0–150 μM; for Fe^3+^, the LOD was found to be 0.10 μM, and the linear range was 0–120 μM. Furthermore, selectivity tests with other metal ions revealed that **ODADA** exhibited superior specificity toward Pb^2+^ and Fe^3+^ ions. The usability of the **ODADA** sensor in practical applications was tested on commercial hair dye samples. The results have shown that this new sensor can also be used in real samples for both Pb^2+^ and Fe^3+^ ions. These results indicate that the new 1,3,4-oxadiazole derivative (**ODADA**) has significant potential as a dual-mode sensor that can be used for the reliable determination of heavy metal ions in environmental and biological samples.

## Supplementary Material



## Notes

Experimental sections; IR, NMR, and HRMS spectra for characterization; UV–vis absorption and fluorescence spectra of the probe **ODADA** (in different solvents, with or without other ions and under different pH conditions); and Job’s and Benesi–Hildebrand plots of the probe **ODADA** with Fe^3+^ (PDF)
