# Supplementary material for: A Novel 1,3,4-Oxadiazole-Based Fast Dual-Mode Sensor for the Detection of Iron and Lead Ions: Synthesis and Photophysical Properties
Source: ACS Omega. 2026 Jun 10;11(24):36117–30. doi: 10.1021/acsomega.6c03258 (PMC13294898; doi:10.1021/acsomega.6c03258)
Supplement: Supplementary file 1 [file ao6c03258_si_001.pdf]

Supporting Information

A novel 1,3,4-oxadiazole-based fast dual-mode sensor for the detection of iron and lead ions: Synthesis and photophysical properties

Abdulrahman Altay[1], Hamza Karakuş[2], Ebru Bozkurt [3,4]*, Muhammet Yildirim[5], Murat Olutas[6]

[1]Ministry of Environment and Climate Change, The Hazardous Chemical & Waste Department, Doha, Qatar
[2] Technology Transfer Application and Research Center, Bolu Abant İzzet Baysal University, Bolu, Türkiye.
[3]Program of Occupational Health and Safety, Vocational College of Technical Sciences, Atatürk University, Erzurum, Türkiye
[4]Department of Nanoscience and Nanoengineering, Graduate School of Natural and Applied Sciences, Atatürk University, Erzurum, Türkiye
[5]Department of Chemistry, Faculty of Arts and Sciences, Bolu Abant İzzet Baysal University, Bolu, Türkiye
[6]Department of Physics, Faculty of Arts and Sciences, Bolu Abant İzzet Baysal University, Bolu, Türkiye

2. Experimental

UV-VIS absorption and fluorescence spectroscopy

The fluorescence quantum yield of probe **ODADA**, both in the absence and presence of metal ions, was determined using the Parker-Rees method, as described by the following equation 1.

$$\Phi_s = \Phi_r \left(\frac{D_S}{D_r}\right)\left(\frac{\eta_s^2}{\eta_r^2}\right)\left(\frac{1-10^{-OD_r}}{1-10^{-OD_s}}\right) \quad (1)$$

Quinine sulfate (Φ_f = 0.55 in 0.5 M H_2SO_4) was used as the reference material for the calculations. In this context, *D* denotes the integrated area under the corrected fluorescence spectrum, *n* represents the refractive index of the solution, and *OD* corresponds to the optical density at the excitation wavelength (λ_{exc} = 350 nm). The subscripts *s* and *r* refer to the sample and reference solutions, respectively [1].

The detection limit values for the identified ions were calculated using data from absorption/fluorescence titration experiments, applying the *3s/k* equation. In this equation, *s* represents the standard deviation of the blank, while *k* denotes the slope of the fitted line obtained from the absorption/fluorescence titration data [2].

Furthermore, the binding stoichiometry was determined using Job's plot analysis, based on the following equation:

$$A_{job} = (1 - X)(A - A_0) \quad (2)$$

In this equation, *X* represents the mole fraction of the detected ion, while A_0 *(F_0)* and *A (F)* correspond to the absorbance (fluorescence) intensities of probe **ODADA** in the absence and presence of ions, respectively [3].

The Benesi-Hildebrand equation was used to obtain the binding constant (K_a).:

$$\frac{1}{A-A_0} = \frac{1}{K_a(A_{max}-A)[M^+]^n} + \frac{1}{A_{max}-A_0} \quad (3)$$

The equation incorporates absorbance (fluorescence) intensity values A_0 (F_0), A (F), and A_{max} (F_{max}), where A_0 (F_0) represents the absorbance (fluorescence) intensity in the absence of a metal ion, A (F) denotes the intensity at a specific metal ion concentration, and A_{max} (F_{max}) corresponds to the intensity at the saturation concentration of the metal ion. Additionally, $[M^+]$ indicates the metal ion concentration, while n represents the binding stoichiometry between the probe and the metal ion [2, 4].

References

[1] Bozkurt E, Gul HI, Ozgun DO. Pyrazoline derived new "off-on-off" fluorescent pH sensors. Optical Materials. 2018;84:550-5.

[2] Kushwaha A, Patil SK, Das D. A pyrene-benzimidazole composed effective fluoride sensor: potential mimicking of a Boolean logic gate. New Journal of Chemistry. 2018;42(11):9200-8.

[3] Zhang Y, Wang G, Zhang J. Study on a highly selective fluorescent chemosensor for Fe3+ based on 1, 3, 4-oxadiazole and phosphonic acid. Sensors and Actuators B: Chemical. 2014;200:259-68.

[4] Kilic H, Bozkurt E. A rhodamine-based novel turn on trivalent ions sensor. Journal of Photochemistry and Photobiology A: Chemistry. 2018;363:23-30.

Analyst	PEService
Date	Friday, November 22, 2024 3:16 PM

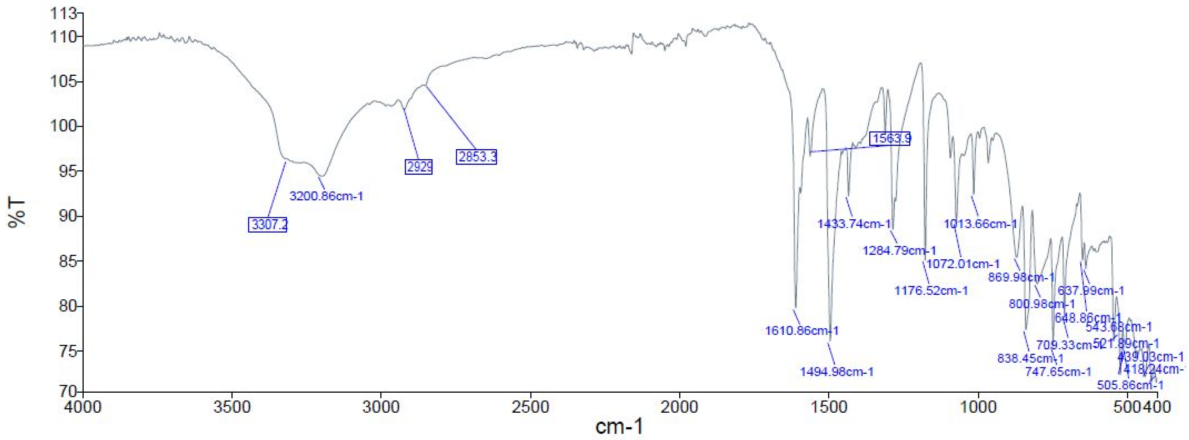

Sample Name	Description	Quality Checks
HKMY106	Sample 145 By PEService Date Friday, November 22 2024	The Quality Checks give rise to multiple warnings for the sample.

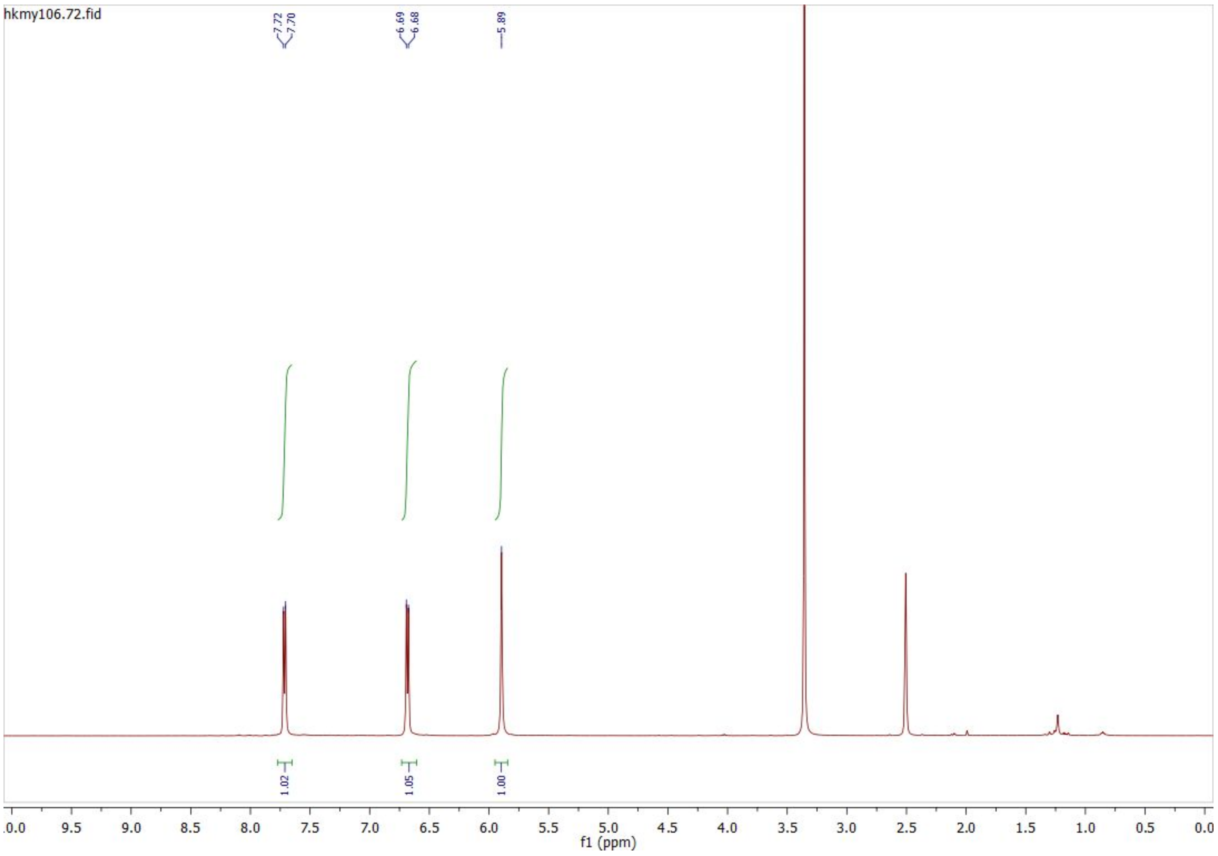

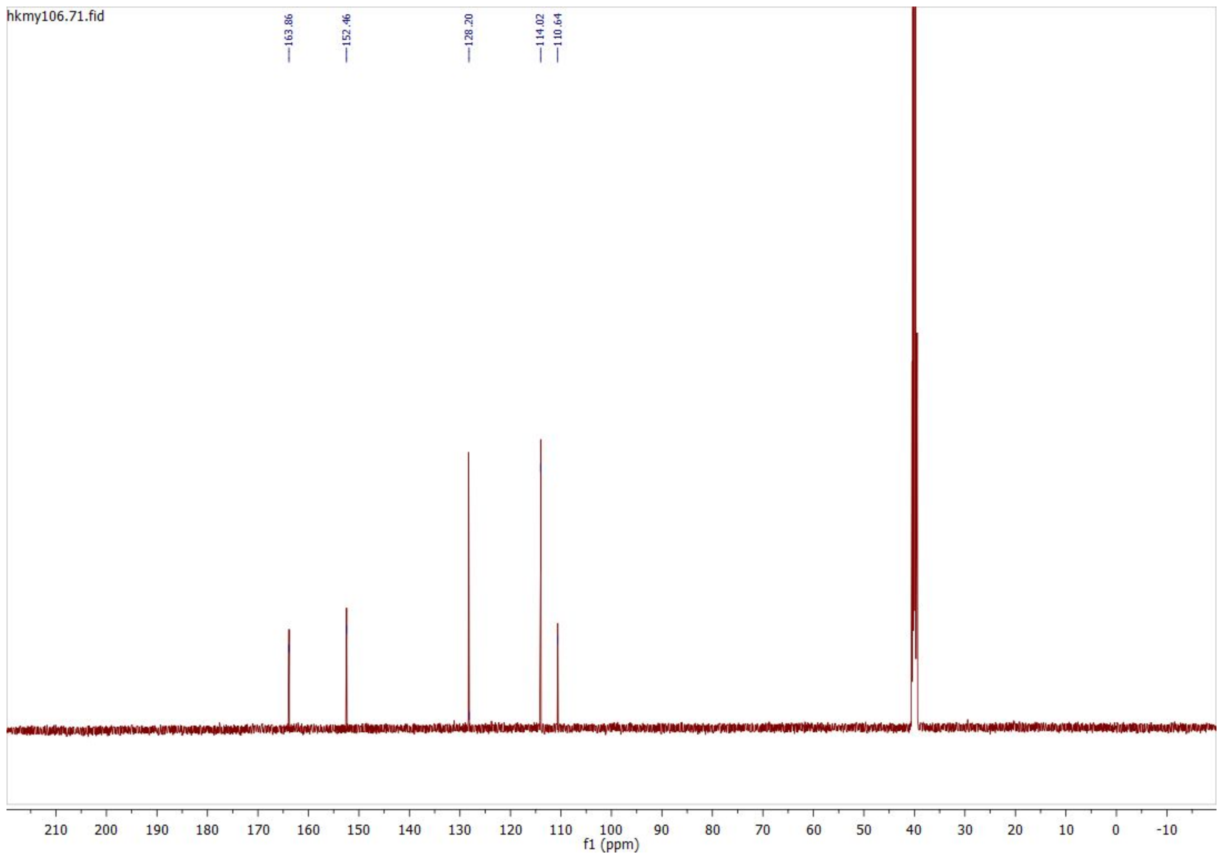

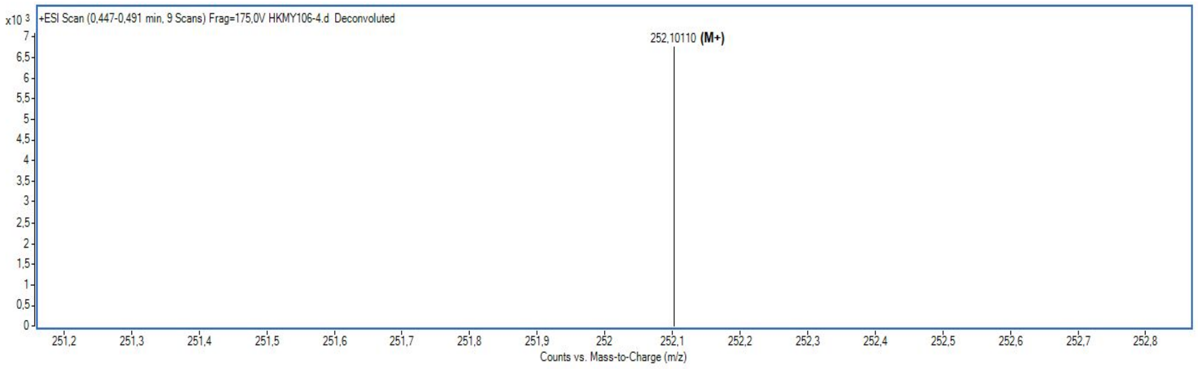

Fig. S1. IR, ^{1}H-NMR, ^{13}C-NMR, HRMS spectra of **ODADA**

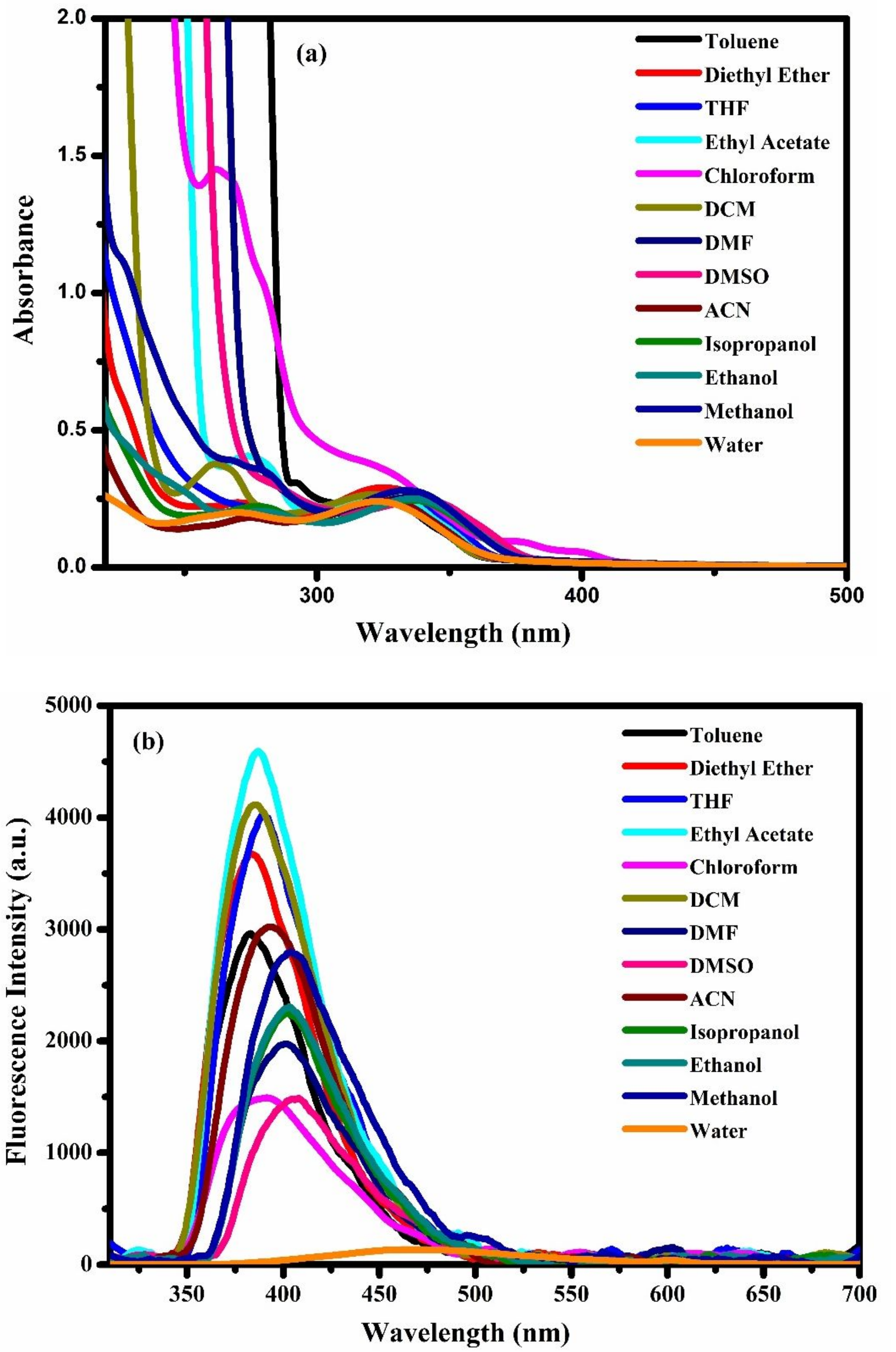

Fig. S2. (a) The UV-vis. absorption and (b) fluorescence spectra of probe **ODADA** (10 μM) in different solvents (λ_{exc}=350 nm)

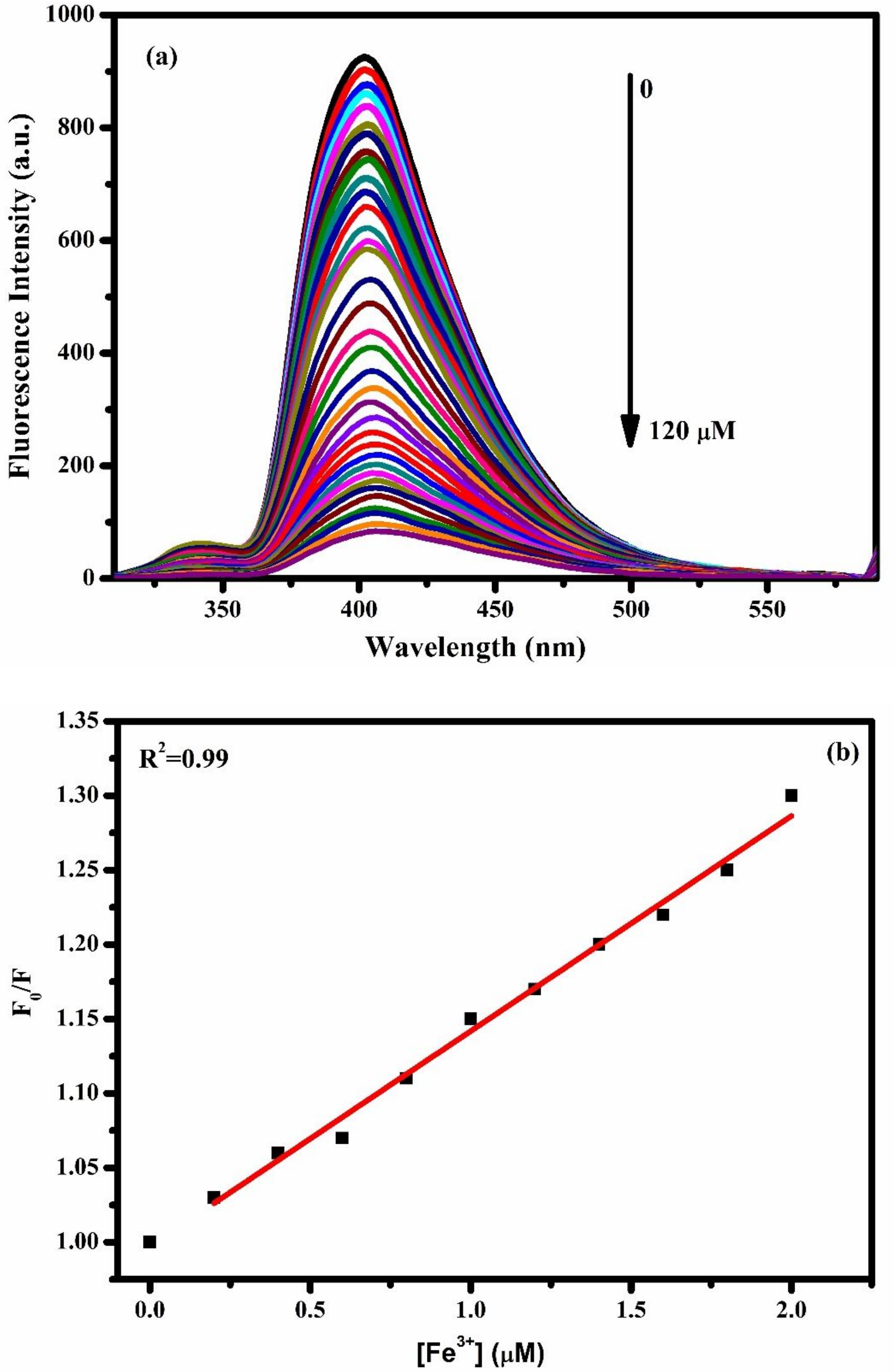

Fig. S3. (a) Fluorescence spectra and (b) variation in absorption intensity of probe **ODADA** (2 μM) with increasing Fe^{3+} ion concentration in ethanol

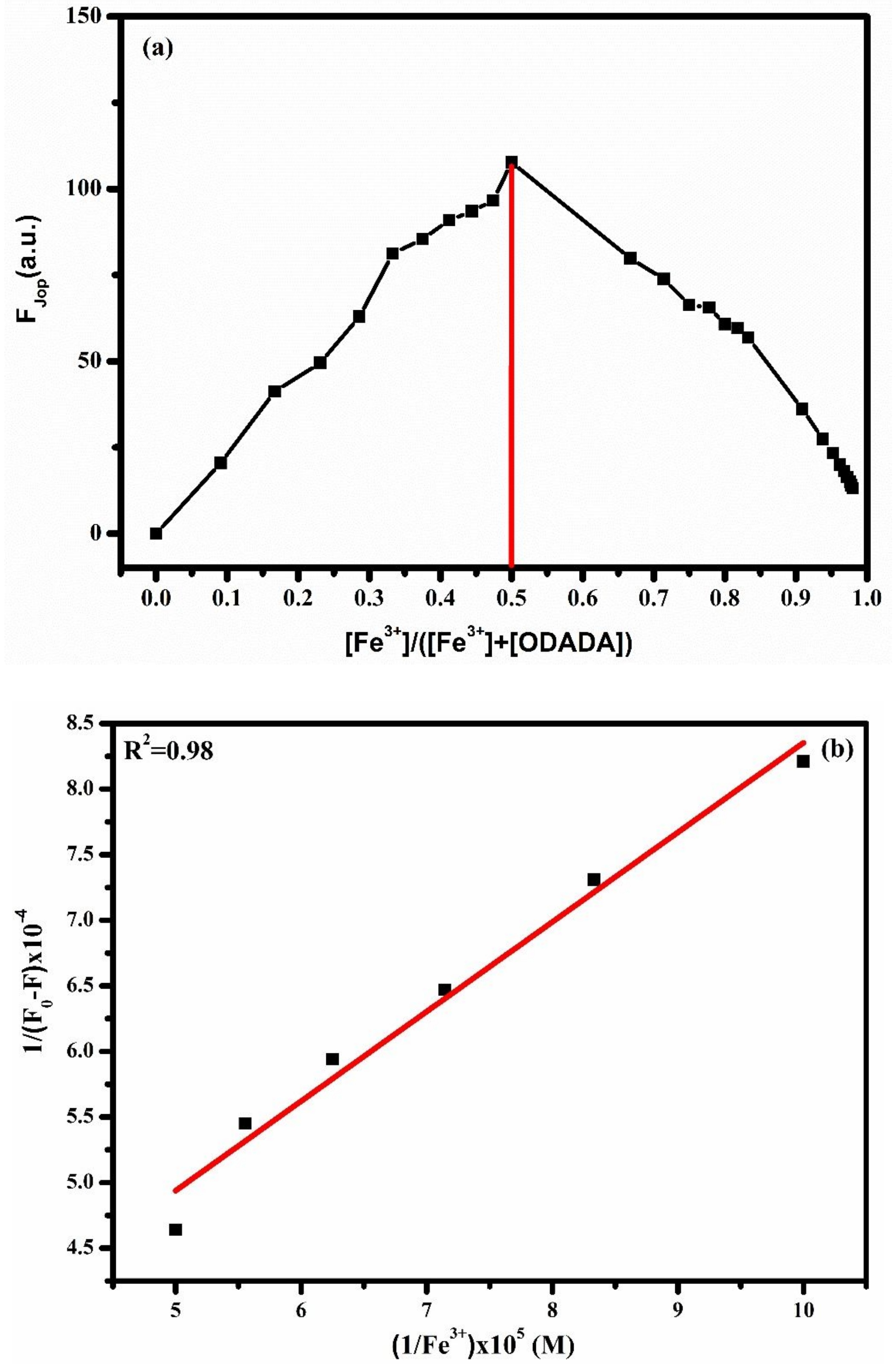

Fig. S4. (a) Job's plot of probe **ODADA** with Fe^{3+} (b) Benesi-Hildebrand plot based on a 1:1 association stoichiometry between Fe^{3+} and probe **ODADA** in ethanol

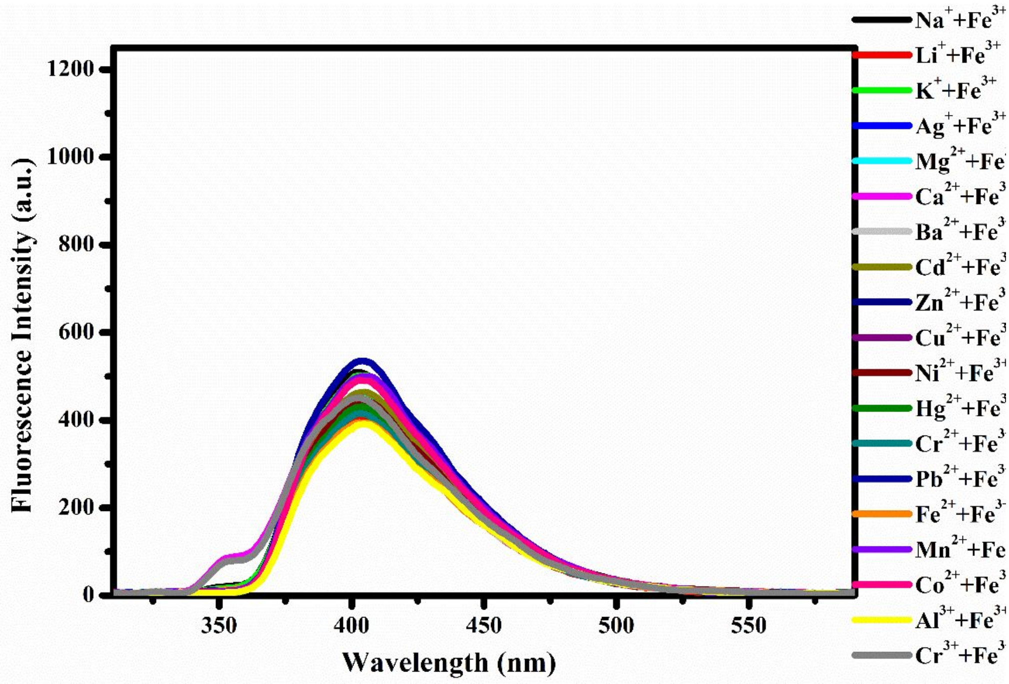

Fig. S5. Fluorescence spectra of probe **ODADA** (2 μM) both studied other ions and Fe^{3+} ion in ethanol

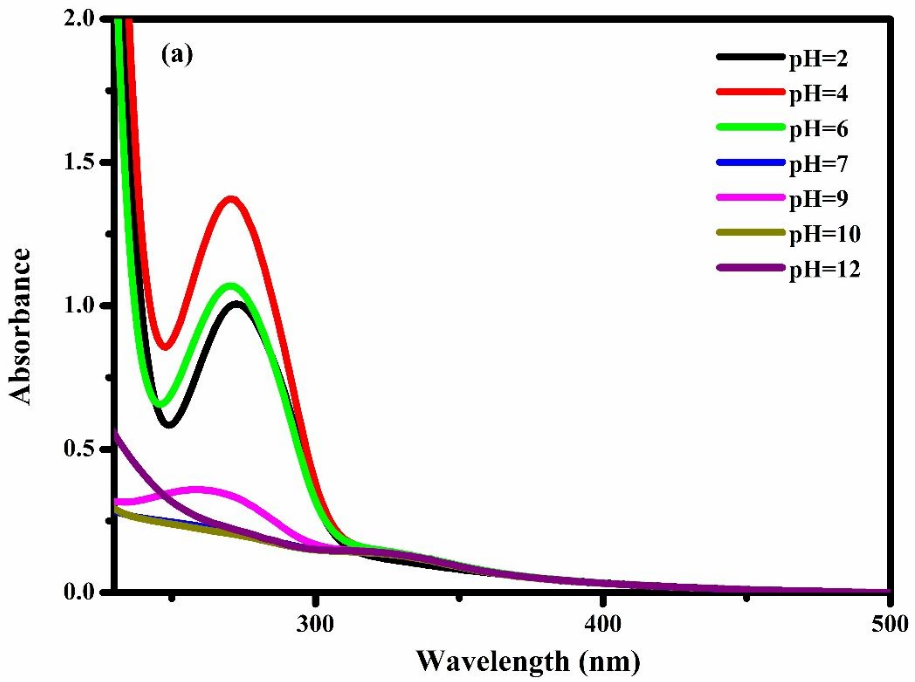

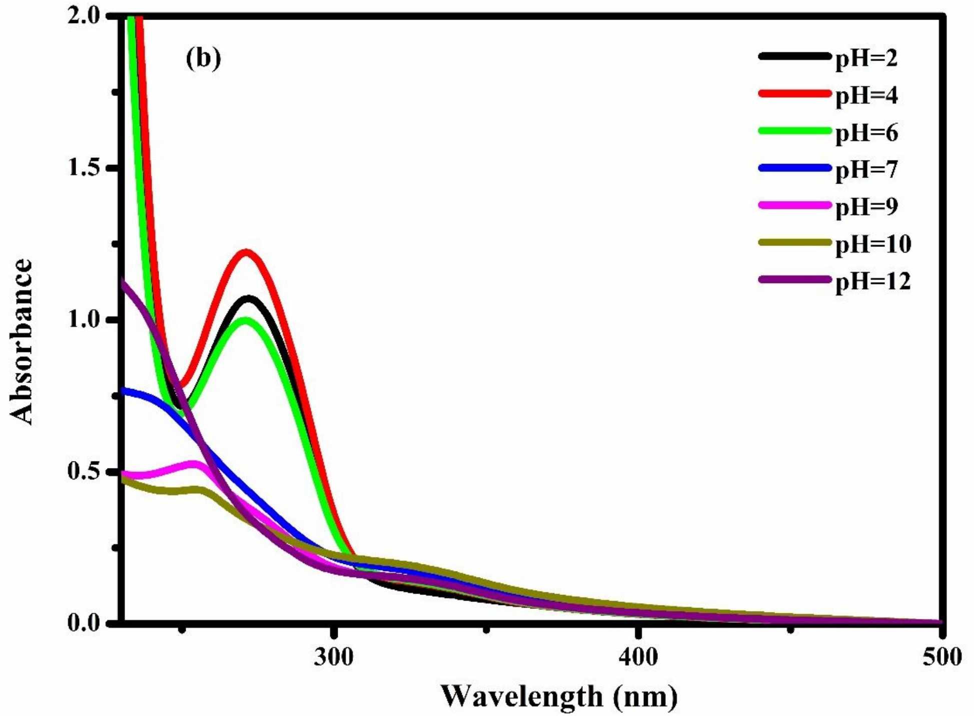

Fig. S6. Absorbance spectra of **ODADA** (a) without Pb^{2+} (b) with Pb^{2+} in different pH

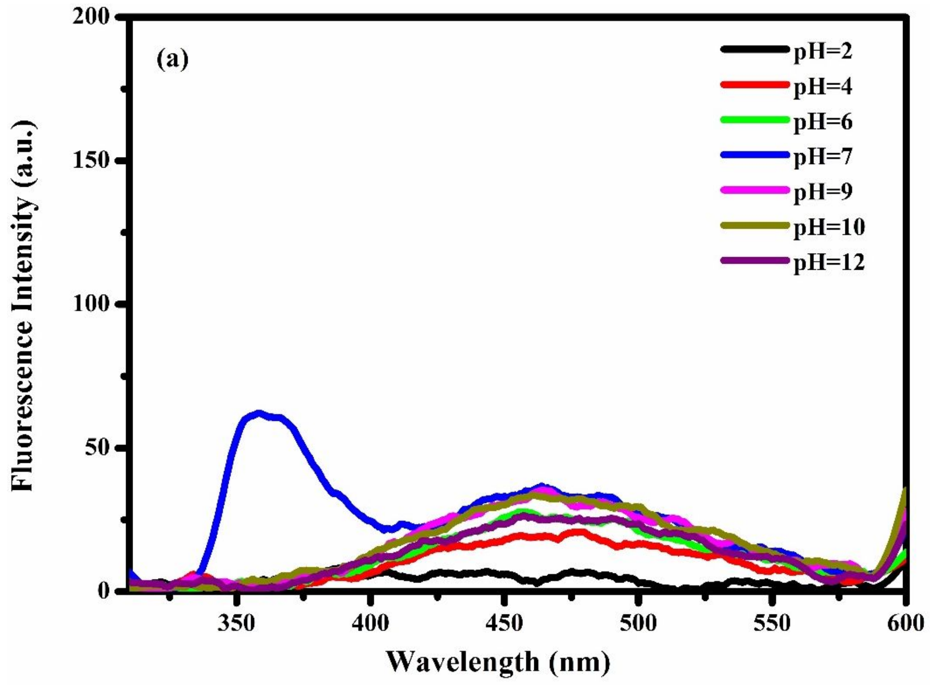

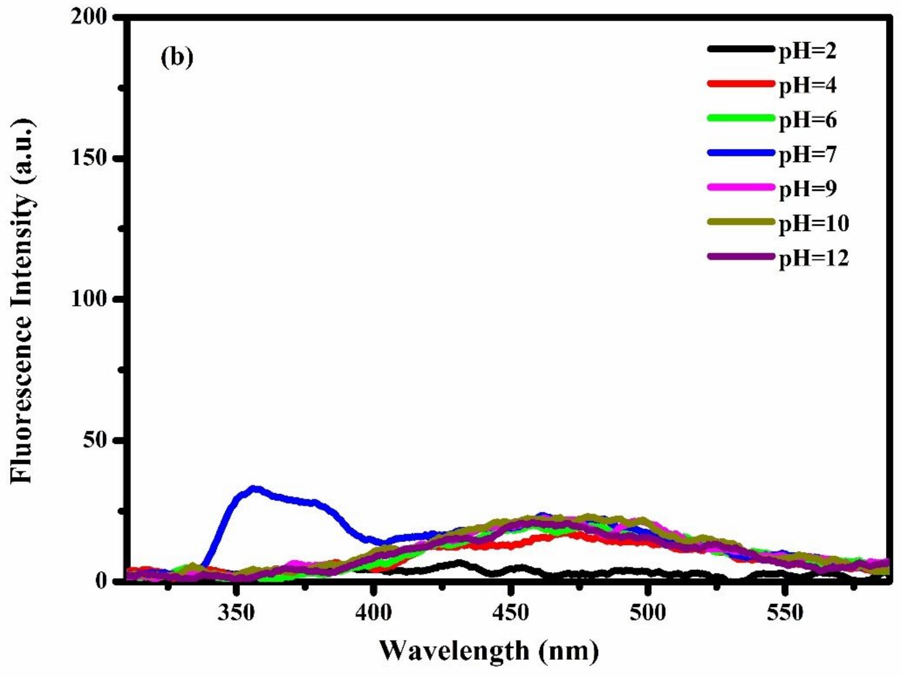

Fig. S7. Fluorescence spectra of **ODADA** (a) without Fe^{3+} (b) with Fe^{3+} in different pH
